# Dataset for the Synthesis of Boron-Dipyrrin Dyes, their fluorescent properties, their interaction with proteins, Triton-X-based surfactants, and micellar clusterization approaches to validation based on fluorescent dyes

**DOI:** 10.1016/j.dib.2022.108464

**Published:** 2022-07-12

**Authors:** Aleksei V. Solomonov, Yuriy S. Marfin, Alexander B. Tesler, Dmitry A. Merkushev, Elizaveta A. Bogatyreva, Elena V. Antina, Evgeniy V. Rumyantsev, Ulyana Shimanovich

**Affiliations:** a Department of Molecular Chemistry and Materials Science, Faculty of Chemistry, Weizmann Institute of Science, Rehovot 7610001, Israel; b Department of Inorganic Chemistry, Ivanovo State University of Chemistry and Technology, 7 Sheremetevskij prosp., Ivanovo 153000, Russian Federation; c Department of Materials Science and Engineering, Friedrich-Alexander University Erlangen-Nuremberg, 7 Martensstrasse, Erlangen 91056, Germany; d G.A. Krestov Institute of Solution Chemistry of the Russian Academy of Sciences, 1 Akademicheskaya St., Ivanovo 153045, Russian Federation; e Ivanovo State Polytechnical University, 20 8th Marta St., Ivanovo 153037, Russian Federation

**Keywords:** Surfactant micelles, Self-assembly, Fluorescent dyes, BODIPY, Synthesis, Solubilization, Encapsulation, Proteins

## Abstract

The data presented here refer to the research article by Aleksei V. Solomonov, Yuriy S. Marfin, Alexander B. Tesler, Dmitry A. Merkushev, Elizaveta A. Bogatyreva, Elena V. Antina, Evgeniy V. Rumyantsev, and Ulyana Shimanovich “Spanning BODIPY fluorescence with self-assembled micellar clusters”, Colloids and Surfaces B: Biointerfaces, 216, 2022, 112532. The present article provides details on optical characterization for a set of *meso*- and *tetra-*substituted boron-dipyrrin (BODIPY) complexes encapsulated inside of self-assembled Triton-X-based micellar coordination clusters (MCCs), based on Triton-X family surfactants. Changes in the optical properties of the BODIPY complexes upon interaction with bovine serum albumin, in a binary mixture of THF:H_2_O and titrated with Triton TX-114, were evaluated. The optical properties and the formation kinetics of the BODIPY-based MCCs and the BODIPY-supported micelle chelator aggregates (MCAs) are presented as well. The presented data provide additional insights into the structural and formation aspects of both the traditional and newly obtained micellar coordination clusters for their future optimization, control, and application. The synthetic procedures for the synthesis of a set of *meso*- and *tetra*-substituted BODIPY complexes and their optical properties in different media are also presented. The research is related to the paper (Solomonov et al., 2022).

**Specifications Table**

**Table:** 

Subject	Chemistry
Specific subject area	Chemistry of materials, surfactants, self-assembly, fluorescent dyes, boron-dipyrrins, optical spectroscopy and microscopy, as well as organic synthesis
Type of data	Tables, Images, Graphs, Figures
How the data were acquired	Absorption spectra were recorded using Agilent-Varian Cary 100 (USA), Aquilon SF-104 (Russian Federation), and SOLAR SM2203 (Belarus) spectrofluorometers. The fluorescence spectra were recorded using the Agilent-Varian Cary Eclipse (USA-Australia) spectrofluorometer with a detector voltage of 600 V, the Horiba Jobin Yvon Fluorolog 3 (USA) spectrofluorometer with a detector voltage of 950 V, and the SOLAR SM2203 (Belarus) spectrofluorometer. Both the absorbance and fluorescence spectra were obtained at 298.2±0.1 K in a thermostatic cell holder supplied with a heat transfer module Peltier PTC-2 (PG Instruments, UK). A Micromed LUM-3 fluorescence microscope, equipped with a ToupCam 5.0 MP CCD digital camera, and an Olympus BX-61 fluorescence microscope, equipped with a QImaging MicroPublisher 3.3 digital camera, were used to obtain the optical and fluorescence microscopy images of the micellar clusters. ^1^H-NMR spectra of the dyes were recorded on an Avance-500 (Bruker, Germany) spectrometer operating at 500 MHz. Elemental analysis was performed on a FLASH EA1112 (TermoQuest, Italy) elemental analyzer. MALDI-TOF analysis was performed on an AXIMA Confidence MALDI-TOF mass spectrometer (Shimadzu, Japan). To obtain fluorescent images in the UV region, side UV illumination was applied using a UV lamp under a 254/365 nm exposure wavelength. Quantum-chemical calculations were carried out using Gaussian G09W, CHARMM. Molecular docking studies were carried out using Autodock 4.2 (part of the MGLtools 1.5.7 package). Molecules were visualized using PyMol 1.7, Chimera 1.15, and Autodesk Fusion 360.
	To estimate the visible size of the clusters, ImageJ 1.6 software was used. ChemDraw Professional 16.0 was used to draw the schemes. Origin Pro 2019b was used to draw the plots.
Data format	Raw Analyzed Filtered
Description of data collection	Absorption spectra were recorded in a 1 cm path length quartz cuvette in the range of 190–1100 nm. Fluorescence measurements were recorded in a wavelength range of 285–800 nm by varying the excitation wavelengths in quartz cuvettes with a light-absorbing layer thickness of 1 × 1 cm (for dye solutions) or of 0.1 × 1 cm (for micellar clusters, a front face detector was used); excitation and emission slits were varied. Optical and fluorescence microscopy images were obtained in dark/bright-field and phase contrast modes. ^1^H-NMR spectra were recorded in CDCl_3_ or CCl_4_ using tetramethylsilane (TMS) as an internal reference.
Data source location	Institution: Department of Molecular Chemistry and Materials Science, Faculty of Chemistry, Weizmann Institute of Science, 7610001 City/Town/Region: Rehovot Country: Israel
Data accessibility	With this article Repository name: Mendeley Data Data identification number: DOI: 10.17632/8g5xwxxfzv.1 Direct URL to data: https://data.mendeley.com/datasets/8g5xwxxfzv/draft?a=ba9638ac-ca14-4ab1-b69e-f1d495ce6d51 Instructions for accessing these data: Through direct URL Cite this dataset: Solomonov, Alexey; Marfin, Yuriy; Tesler, Alexander; Merkushev, Dmitry; Banakova, Elizaveta; Antina, Elena; Rumyantsev, Evgeniy; Shimanovich, Ulyana (2022), “Dataset for the Synthesis of Boron-Dipyrrin Dyes, their Fluorescent Properties, their Interaction with Proteins, Triton-X-Based Surfactants, and Micellar Clusterisization Approaches to Validation based on Fluorescent Dyes”, Mendeley Data, V1, doi: 10.17632/8g5xwxxfzv.1
Related research article	Aleksei V. Solomonov, Yuriy S. Marfin, Alexander B. Tesler, Dmitry A. Merkushev, Elizaveta A. Bogatyreva, Elena V. Antina, Evgeniy V. Rumyantsev, and Ulyana Shimanovich Spanning BODIPY fluorescence with self-assembled micellar clusters, Colloids and Surfaces B: Biointerfaces, 216, 2022, 112532. 10.1016/j.colsurfb.2022.112532.

**Value of the Data**

• The presented data provide insights into the structural and formative aspects as well as the optical characteristics for a set of newly formed self-assembled micellar coordination clusters. This is based on applying fluorescent boron-dipyrrin and non-ionic Triton-X family surfactants as well as understanding the behavior of these fluorescent dyes in different media.
• The data are relevant to the research and development of new self-assembled systems; the investigation of the optical properties of the fluorescent dyes, especially boron-dipyrrins, is of great value to researchers in related fields.
• An application of fluorescent compounds as a part of micellar-based self-assembled systems may highlight the peculiarities of micellar cluster formation.
• The data presented highlight the nature of micellar self-assembled systems based on non-ionic surfactant formation and their properties for their future optimization, control, and application, as well as the development and application of fluorescent dyes in self-assembled systems.
• The data summarize the synthesis and optical properties of a set of *meso-* and *tetra-*substituted BODIPY dyes.

## 1 Data Description

*Table of Contents*

1. *Meso- and tetra*-substituted BODIPY synthesis
2. BODIPY dyes’ optical characteristics
3. Spectra of BODIPY dyes in a binary mixture of THF:H_2_O 5:95 (v/v)
4. Validation of the MCC approach
5. TX-100-based MCCs
6. CLSM images of BODIPY-encapsulated MCCs
7. CLSM images of BODIPY-supported MCAs
8. Spectra of BODIPYs titrated with TX-114
9. Changes in the fluorescence spectra of the dyes in TX-114, MCAs, and MCCs
10. Kinetics studies for mAB, mPB, and TPB fluorescence during MCA formation
11. Interaction of the *meso*-BODIPY dyes with BSA protein
12. Structure and description of the data in the Mendeley repository.

*1. Meso- and tetra-substituted BODIPY synthesis*

**Scheme 1:**
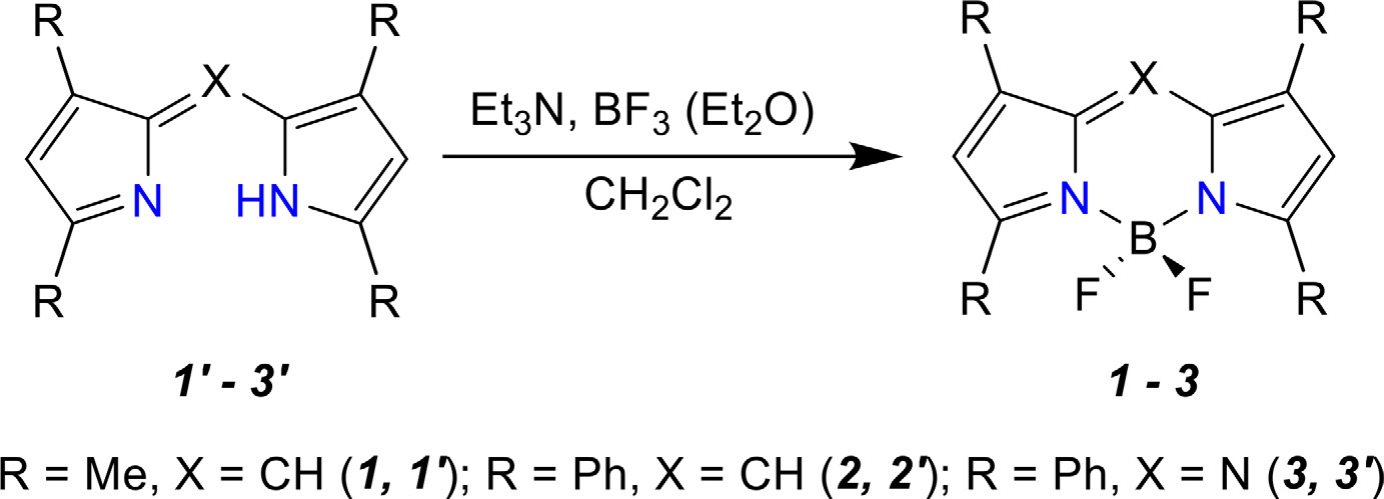
Synthetic route for the tetra-substituted dyes.

*Tetra*-substituted BODIPYs: Series of *tetra*-substituted BODIPYs, 3,3′,5,5′-Tetramethyl-2,2′-dipyrrolylmethene difluoroborate (*tetra*-Me-BODIPY, TMB); 3,3′,5,5′-Tetraphenyl-2,2′-dipyrrolylmethene difluoroborate (*tetra*-Ph-BODIPY, TPB), 3,3′,5,5′-Tetraphenyl-*meso-aza*-2,2′-dipyrrolylmethene difluoroborate (*tetra*-Ph-*aza*-BODIPY, aTPB) were synthesized via our previously reported procedures [2], [3], [4], Scheme 1.

*Tetra-substituted BODIPYs*:

3,3′,5,5′-Tetramethyl-2,2′-dipyrrolylmethene hydrobromide (1’) was synthesized, purified, and identified according to our previously reported procedure [2]. Briefly, 2 mL of concentrated HBr (48 wt. % in H_2_O, ≥99.99%, Sigma-Aldrich, USA) was added to a stirred solution of 1.2 g (12.6 mmol) of 2,4-dimethylpyrrole (97%, Sigma-Aldrich, USA) and 1.55 g (12.6 mmol) of 2-formyl-3,5-dimethylpyrrole (95%, Sigma-Aldrich, USA) in 20 mL of methanol at ambient temperature. After the mixture was stirred for 2 h, the precipitate was separated via filtration, then washed with methanol, diethyl ether (HPLC, ≥99.9%, Sigma-Aldrich, USA), and dried in air. The yield was 2.4 g (67.7%). ^1^H NMR (δ, ppm): 13.12 (br s, 2H, NH), 7.09 (s, 1H, ms-H), 6.19 (s, 2H, 4,4′-H), 2.71 (s, 6H, CH_3_), 2.37 (s, 6H, CH_3_). For C_13_H_17_N_2_Br anal. calculated (%): C, 55.70; H, 6.12; N, 10.00. Measured (%): C, 55.25; H, 6.05; N, 9.89. The synthesis of the required pyrroles is described in detail in our previous reports [2].

3,3′,5,5′-Tetraphenyl-2,2′-dipyrrolylmethene (2’) was synthesized using the procedure described in previous reports [3]. Briefly, a mixture of 0.44 g (2.02 mmol) of 2,4-diphenylpyrrole, 0.5 g (2.02 mmol) of 2-formyl-3,5-diphenylpyrrole (95%, Sigma-Aldrich, USA), 10 mL of acetic acid (ACS reagent, ≥99.7%, Sigma-Aldrich, USA), and 2.5 mL of acetic anhydride (ACS reagent, ≥98.0%, Sigma-Aldrich, USA) was heated under reflux conditions for 1 h. The solution turns crimson then precipitates. The precipitate was filtered, washed with methanol, then dried under air. The acetic acid solution was poured into 200 mL of Milli-Q water; the precipitate was filtered, dried, and chromatographed on silica gel (L 100/250, chloroform as an eluent). The yield was 0.7 g (77.2%). The absorption spectrum was λ_max_ = 532, 295 nm (benzene). ^1^H NMR (δ, ppm): 14.35 (br s, 1H, NH), 7.94 – 8.03 (m, 8H, o-H–Ph), 7.42–7.60 (m, 12H, m,p-H-Ph), 6.99 (s, 2H, 4,4′-H), 6.76 (s, 1H, ms-H). For C_33_H_24_N_2_ anal. calculated (%): C, 88.36; H, 5.39; N, 6.25. Measured (%): C, 88.51; H, 5.18; N, 6.12.

2,4-Diphenylpyrrole, required for the synthesis of (2), was obtained as reported previously [4]. 2-formyl-3,5-diphenylpyrrole was prepared by the formylation of 2,4-diphenylpyrrole in DMF medium as follows: phosphorus oxychloride (1.0 mL, 10.92 mmol) was added dropwise to a stirred solution of 1.0 g (4.56 mmol) of 2,4-diphenylpyrrole in 5 mL of dry DMF and at a temperature below 10 °С. This mixture was stirred for 1.5 h at ambient temperature, poured into 100 mL of Milli-Q water, and alkalized with 20% sodium hydroxide solution (∼2.7 g of NaOH) at 10 °С. The precipitate was filtered, washed with Milli-Q water, and dried at ambient temperature. The yield was 1.2 g (4.45 mmol, 97.5%).

3,3′,5,5′-Tetraphenyl-ms-aza-2,2′-dipyrrolylmethene (3’). 1-Nitro-2,4-diphenylbutanone-4 (3 g, 3.71 mmol) and 25 g of ammonium acetate (BioUltra, ≥99.0%, Sigma-Aldrich, USA) were placed into a 100 mL round bottom flask and heated in an oil bath at 190 °С, for 2 h. Then the mixture was cooled and diluted with Milli-Q water; the precipitate was filtered, washed several times with Milli-Q water, and dried under air. The yield was 2.35 g (5.22 mmol, 70.2%). ^1^H NMR (δ, ppm): 8.04 – 8.15 (m, 8H, o-H–Ph), 7.44 – 7.58 (m, 12H, m,p-H–Ph), 7.07 (s, 2H, 4,4′H). For C_32_H_23_N_3_ anal. calculated (%): C, 85.50; H, 5.16; N, 9.35. Measured (%): C, 85.59; H, 5.26; N, 9.24.

1-Nitro-2,4-diphenylbutanone-4 is required for the synthesis of 3,3′,5,5′-tetraphenyl-ms-aza-2,2′-dipyrrolylmethene and was obtained as follows: a solution of 10.0 g (48.0 mmol) of chalcone (97%, Sigma-Aldrich, USA), 10.4 mL (0.193 mol) of nitromethane (ReagentPlus®, ≥99.0%, Sigma-Aldrich, USA), and 15.0 mL (0.145 mol) of diethylamine (99.5%, Sigma-Aldrich, USA) in 100 mL of methanol was heated for 24 h under reflux conditions; then the methanol was distilled using a rotary evaporator, and the product was recrystallized in methanol. The yield was 8.14 g (30.24 mmol, 63%). mp 97–99°C.

*3,3′,5,5′-Tetramethyl-2,2′-dipyrrolylmethene difluoroborate (1, tetra-Me-BODIPY, TMB)* was prepared according to the previous reports [2]. Triethylamine (1.59 mL, 11.5 mmol, ≥99.5%, Sigma-Aldrich, USA) was added to a stirred solution of 0.322 g (1.14 mmol) of 3,3′,5,5′-tetramethyl-2,2′-dipyrrolylmethene hydrobromide dissolved in 40 mL of absolute dichloromethane (≥99.8%, Sigma-Aldrich, USA) at ambient temperature; then 1.44 mL (11.5 mmol) of boron trifluoride etherate (for synthesis, Sigma-Aldrich, USA) was immediately added. This mixture was stirred for 3 h and washed three times with Milli-Q water; then, the organic layer was separated by drying under reduced pressure. The obtained material was dissolved in absolute dichloromethane and chromatographed on silica gel (L 100/250). Next, the solvent was evaporated; the complex was precipitated with methanol, filtered, and dried under air at ambient temperature. The yield was 0.263 g (1.06 mmol, 92.9%). The absorption spectrum (λ_max_, nm (log ε)) is 508 (4.95), 363 (3.78) (CHCl_3_); 503 (4.85), 364 (3.70) (DMF). ^1^H NMR (500 MHz, CDCl_3_, δ, ppm): 7.07 (s, 1H, ms-H), 6.07 (s, 2H, 4,4′-H), 2.55 (s, 6H, CH_3_), 2.27 (s, 6H, CH_3_). For C_13_H_15_BF_2_N_2_ anal. calculated (%): C, 62.94; H, 6.09; N, 11.29. Measured (%): C, 62.83; H, 6.00; N, 11.18. Difluoroborates 2′ and 3′ were obtained using a similar procedure.

*3,3′,5,5′-Tetraphenyl-2,2′dipyrrolylmethene difluoroborate (2, tetra-Ph-BODIPY, TPB)* was obtained from 3,3′,5,5′-tetraphenyl-2,2′-dipyrrolylmethene. The yield was 79.5%. The absorption spectrum (λ_max_, nm (log ε)) is 569 (4.36), 288 (4.26) (CHCl_3_); 569 (4.41), 286 (4.33) (DMF). ^1^H NMR (500 MHz, CDCl_3_), δ, ppm): 7.43–7.58 (m, 8H, o-H-Ph), 7.17–7.40 (m, 12H, m,p-H-Ph), 6.76 (s, 2H, 4,4′-H), 5.40 (s, 1H, ms-H). For C_33_H_23_BF_2_N_2_ anal. calculated (%): C, 79.85; H, 4.67; N, 5.64. Measured (%): C, 79.07; H, 4.48; N, 5.32.

*3,3′,5,5′-Tetraphenyl-ms-aza-2,2′-dipyrrolylmethene difluoroborate (3, tetra-Ph-aza-BODIPY, aTPB)* was prepared from 3,3′,5,5′-tetraphenylmsaza-2,2′dipyrrolylmethene. The yield was 85.6%. The absorption spectrum (λ_max_, nm (log ε)) is 649 (4.92), 476 (4.00), 309 (4.46) (CHCl_3_); 654 (4.97), 481 (4.04), 307 (4.46) (DMF). ^1^H NMR (500 MHz, CDCl_3_, δ, ppm): 8.04 – 8.15 (m, 8H, o-H-Ph), 7.44–7.58 (m, 12H, m,p-H-Ph), 7.07 (s, 2H, 4,4′-H). For C_32_H_22_BF_2_N_3_ anal. calculated (%): C, 77.28; H, 4.46; N, 8.45. Measured (%): C, 76.85; H, 4.22; N, 8.32.

*Meso-substituted BODIPYs*

**Scheme 2:**
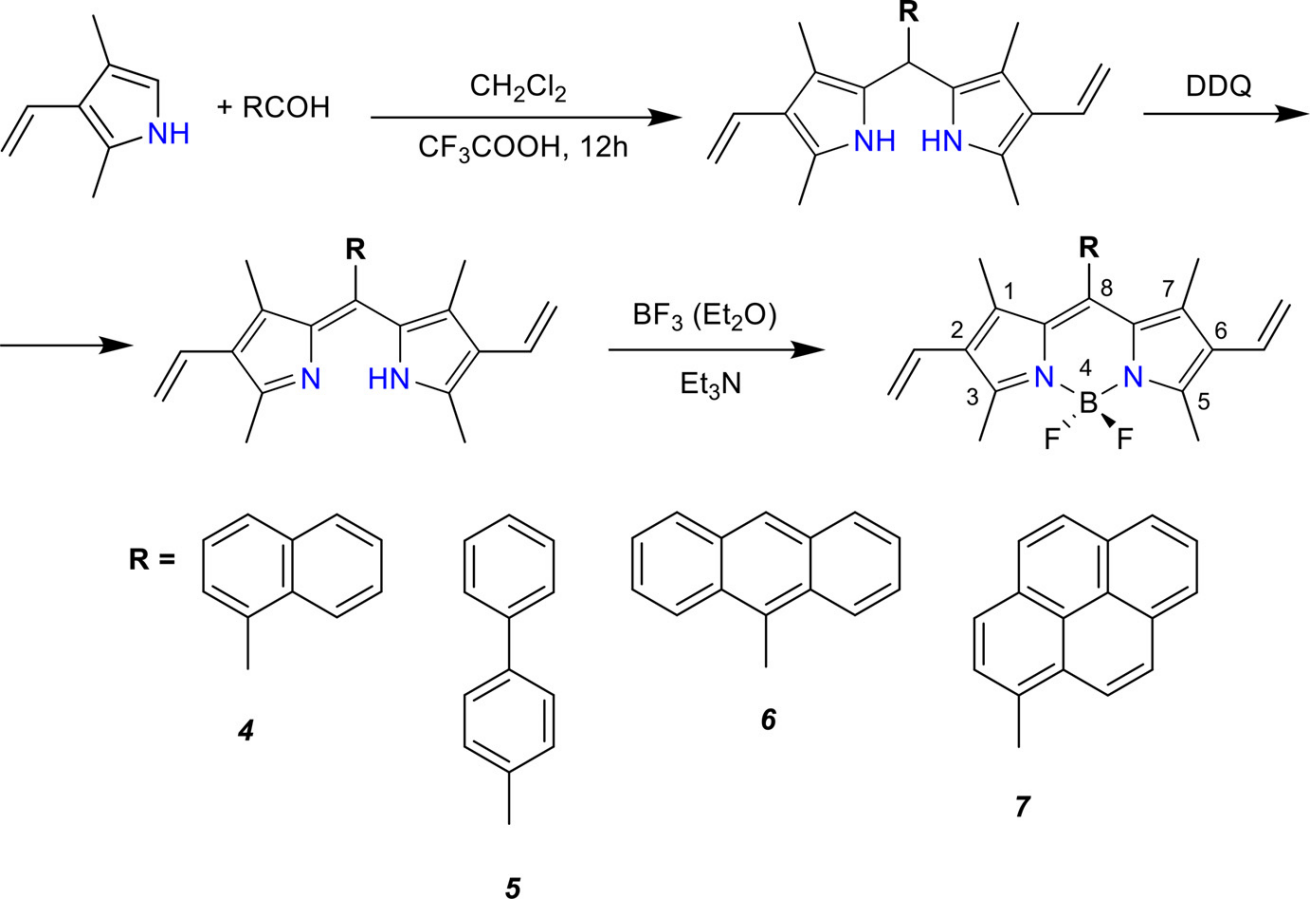
Synthetic route to the meso-substituted dyes.

Meso-substituted BODIPYs: A set of meso-substituted BODIPYs (4,4-difluoro-8-naphthyl-1,3,5,7-tetramethyl-2,6-diethyl-4-boron-3a,4a-diaza-s-indacene, *meso*-Naphtyl-BODIPY, mNpB), (4,4-difluoro-8-diphenyl-1,3,5,7-tetramethyl-2,6-diethyl-4-boron-3a,4a-diaza-s-indacene*, meso*-Diphenyl-BODIPY, mDiPB), (4,4-difluoro-8-antryl-1,3,5,7-tetramethyl-2,6-diethyl-4-boron-3a,4a-diaza-s-indacene, *meso*-Antryl-BODIPY, mAB), and (4,4-difluoro-8-pyrenyl-1,3,5,7-tetramethyl-2,6-diethyl-4-boron-3a,4a-diaza-s-indacene*, meso*-Pyrenyl-BODIPY, mPB) were synthesized using the procedures described in our previous reports [5], Scheme 2.

An aldehyde (naphthyl, diphenyl, anthryl, or pyrenyl, 0.002 moles) and 2,4-dimethyl-3-ethylpyrrole (0.004 moles, 97%, Sigma-Aldrich, USA) were dissolved in 50 mL of absolute dichloromethane. One drop of trifluoroacetic acid (ReagentPlus®, 99%, Sigma-Aldrich, USA) was added, and the solution was stirred for 12 h at room temperature. Then, 0.002 mole of 2,3-dichloro-5,6-dicyano-1,4-benzoquinone (DDQ, 98%, Sigma-Aldrich, USA) was added, along with additional stirring for 20 min. Next, boron trifluoride diethyl etherate (4 mL) and triethylamine (4 mL) were added and the solution was stirred for 30 min. The appearance of an intense fluorescence signal was observed at this stage. The formation of intermediates and BODIPY products was controlled by changing the electron absorption spectra of the solution at every stage. After the synthesis, the reaction mixture was washed with Milli-Q water, dried using anhydrous MgSO_4_ (ReagentPlus®, 99.5%, Sigma-Aldrich, USA), filtered, and evaporated. The obtained compound was purified by silica gel chromatography (eluent – С_2_H_2_Cl_2_/hexane) to obtain analytically pure samples. Here, the one-pot synthesis allowed us to reduce the losses associated with the isolation and purification of intermediates and to increase the yields of BODIPY.

*4,4-difluoro-8-naphthyl-1,3,5,7-tetramethyl-2,6-diethyl-4-boron-3a,4a-diaza-s-indacene (4, mNpB)* was synthesized according to the general synthesis procedure (see Scheme S2) using 1-naphthaldehyde (95%, Sigma-Aldrich, USA) as a precursor. The obtained product consisted of dark red crystals (yield = 15.6%). ^1^H NMR (500 MHz, CCl_4_): δ 1.16(T,6H), 1.76 (Q, 6H), 2.23 (Q, 4H), 2.75 (S,6H), 7.19 (S, 3H), 7.46 (Q, 2H), 7.97 (Q, 2H). MALDI-TOF: calculated ([C_27_H_29_BF_2_N_2_]^+^) m/z = 430.35, detected m/z = 429.92. Anal. calculated for: C_27_H_29_BF_2_N_2_: C, 75.36; H, 6.79; N, 6.51. Measured: C, 75.13; H, 6.32; N, 6.50.

*4,4-difluoro-8-diphenyl-1,3,5,7-tetramethyl-2,6-diethyl-4-boron-3a,4a-diaza-s-indacene (5, mDiPB)* was synthesized according to the general synthesis procedure (see Scheme S2) using diphenyl-4-carboxaldehyde (99%, Sigma-Aldrich, USA) as a precursor. The obtained product consists of bright green crystals (yield = 24.3 %). ^1^H NMR (500 MHz, CCl_4_): δ 1.15 (T, 6H), 1.78 (Q, 6H), 2.27 (Q, 4H), 2.75 (S, 6H), 6.56 (S, 2H), 7.34 (D, 4H), 7,46 (S, 2H), 8.01 (S, 1H). MALDI-TOF: calculated ([C_29_H_31_BF_2_N_2_]^+^) m/z = 456.39, detected m/z = 458.13. Anal. calculated for: C_29_H_31_BF_2_N_2_: C, 76.32; H, 6.85; N, 6.14. Measured: C, 76.28; H, 6.75; N, 6.19.

*4,4-difluoro-8-antryl-1,3,5,7-tetramethyl-2,6-diethyl-4-boron-3a,4a-diaza-s-indacene (6, mAB)* was synthesized according to the general synthesis procedure (see Scheme S2) using 9-anthracenecarboxaldehyde (97%, Sigma-Aldrich, USA) as a precursor. The obtained product consisted of dark green crystals (yield = 19.4%). ^1^H NMR (500 MHz, CCl_4_): δ 1.16(T,6H), 1.80 (Q, 6H), 2.25 (Q, 4H), 2.73 (S, 6H), 7.15 (S, 3H), 7.46 (Q, 4H), 7.93 (Q, 2H). MALDI-TOF: calculated ([C_31_H_31_BF_2_N_2_]^+^) m/z = 480.41, detected m/z = 479.95. Anal. calculated for: C_31_H_31_BF_2_N_2_: C, 77.51; H, 6.50; N, 5.83. Measured: C, 77.50; H, 6.28; N, 5.79.

*4,4-difluoro-8-pyrenyl-1,3,5,7-tetramethyl-2,6-diethyl-4-boron-3a,4a-diaza-s-indacene (7, mPB)* was synthesized according to the general synthesis procedure (see Scheme S2) using 1-pyrenecarboxaldehyde as a precursor (99%, Sigma-Aldrich, USA. The obtained product consisted of bright green crystals (yield = 25.8%). ^1^H NMR (500 MHz, CCl_4_): δ 1.17 (T, 6H), 2.07 (Q, 6H), 2.23 (S, 4H), 2.76 (S, 6H), 6.53 (S, 2H), 7.30 (D, 3H), 7.41 (S, 2H), 7.90 (S, 2H). MALDI-TOF: calculated ([C_33_H_31_BF_2_N_2_]^+^) m/z = 504.43, detected m/z = 504.01. Anal. calculated for: C_33_H_31_BF_2_N_2_: C, 78.58; H, 6.19; N, 7.53. Measured: C, 78.32; H, 6.15; N, 7.51.

The Mendeley repository for this section contains the full set of data for each dye (path: “Root – Data in Brief main dataset – Data for BODIPY synthesis (Section 1. Meso- and tetra-substituted BODIPY synthesis)”): ^1^H-NMR.xlsx; Elemental analysis.xlsx; MALDI-TOF.xlsx; UV-VIS Spectra.xlsx.

*2. BODIPY dyes’ optical characteristics*

BODIPY (boron-dipyrromethene, 4,4-difluoro-4-bora-3a,4a-diaza-*s*-indacene boron fluoride complex) dyes were discovered by Treibs and co-workers in 1968. These compounds were obtained from a wide family of dipyrromethenes (DPM) by complexing them with boron trifluoride etherate.

**Fig. 1:**
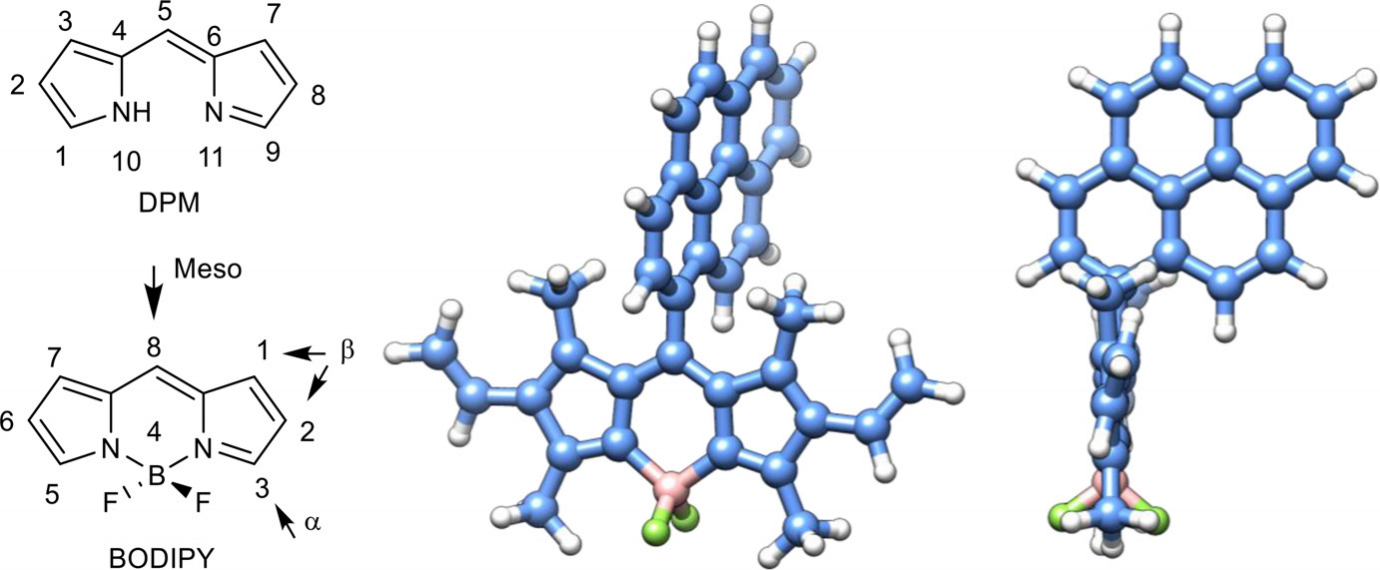
Dipyrromethene (DPM), boron-dipyrromethene (BODIPY) skeleton, their IUPAC numbering scheme (left), and a quantum-chemically optimized molecule of mPB at different angles of view (center and right). The BODIPY structure was optimized by Gaussian G09 software using the DFT/B3LYP/6-311G (d,p) method. Molecule visualization – Chimera 1.16, structural formulas – ChemDraw 2016.

Typically, the BODIPY skeleton core (Fig. 1) is planar and rigid, whereas the fluorine atoms are located on different sides of the core plane. Owing to the aromatic backbone, BODIPY dyes tend to form assemblies and aggregates, which have been studied and discussed extensively in recent years. Along with the other fluorescent dyes, such as fluorescein-, rhodamine-like ones, and others, BODIPYs are widely used in developing fluorescent probes due to the following characteristics:

(1) There are sharp and narrow absorption and fluorescence peaks in the visible/near-infrared regions.
(2) There are large absorption coefficients (sometimes more than 100000), high fluorescence quantum yields (close to 100%), and reasonably long singlet state lifetimes.
(3) The synthesis procedures are simple.
(4) It is easy to tune their photophysical characteristics by changing the substituents in the BODIPY core.
(5) There is a wide range of different applications.

Owing to these properties, the BODIPY dyes are widely used as fluorescent probes in biology for fluorescence imaging of biomolecules in living cells, as sensors on cations, anions, and pH, drug delivery agent trackers, fluorescent switches, parts of electroluminescent films, organic light-emitting devices, laser dyes, and light harvesters, as well as sensitizers for solar cells.

By introducing substituents with different electron densities into the structure of the BODIPY core, it is possible to change the photophysical properties of BODIPYs by manipulating the conjugation length or by chemical modification at different positions of the dye core structure. BODIPY dyes are relatively easy to functionalize in all positions to obtain various BODIPY derivatives with desired properties. Functional groups can be introduced into various positions of BODIPY: directly into the *meso* group; into a mesoaryl group; into pyrrole carbon atoms of the BODIPY core, as well as into the B(III) coordination center. However, often the BODIPY properties can be easily and effectively modulated by simple and facile structural modifications at the *α, β,* and *meso* positions via different synthetic strategies.

Recently, several reviews have appeared, describing various aspects of BODIPYs that contain a functionalized aryl group at the *meso* position. This is one of the most suitable positions for substituted BODIPY dye synthesis due to the simple way of obtaining them from various aromatic aldehydes. The obtained compounds are widely used for fabrication of complex light-harvesting arrays, photonic wires, and opto-electronic gates. The *meso*-aryl functionalized BODIPYs can be easily prepared by following a well-established traditional Lewis acid catalyzed approach.

Azadipyrromethene ligand, in which the –CH_2_– bridge group at the *meso* position was replaced by a nitrogen atom (also called *aza*-BODIPYs), was first reported in 1943 by M. Rogers; later, these compounds were found to be useful in many applications. Often, owing to their near-infrared (NIR) absorption, an adjustable wavelength of fluorescence/absorption peak, and a high molar extinction coefficient, *aza*-BODIPYs have been widely applied in NIR dyes, fluorescent chemosensors, sensitized solar cells, photothermal therapy (PTT), and photodynamic therapy (PDT). The effect of near infrared emission can also be tuned via the symmetrical introduction of *e.g.*, aryl substituents into the 1,3,5,7 positions to move the emission wavelength to bring it to a therapeutic window of 650−900 nm.

**Fig. 2:**
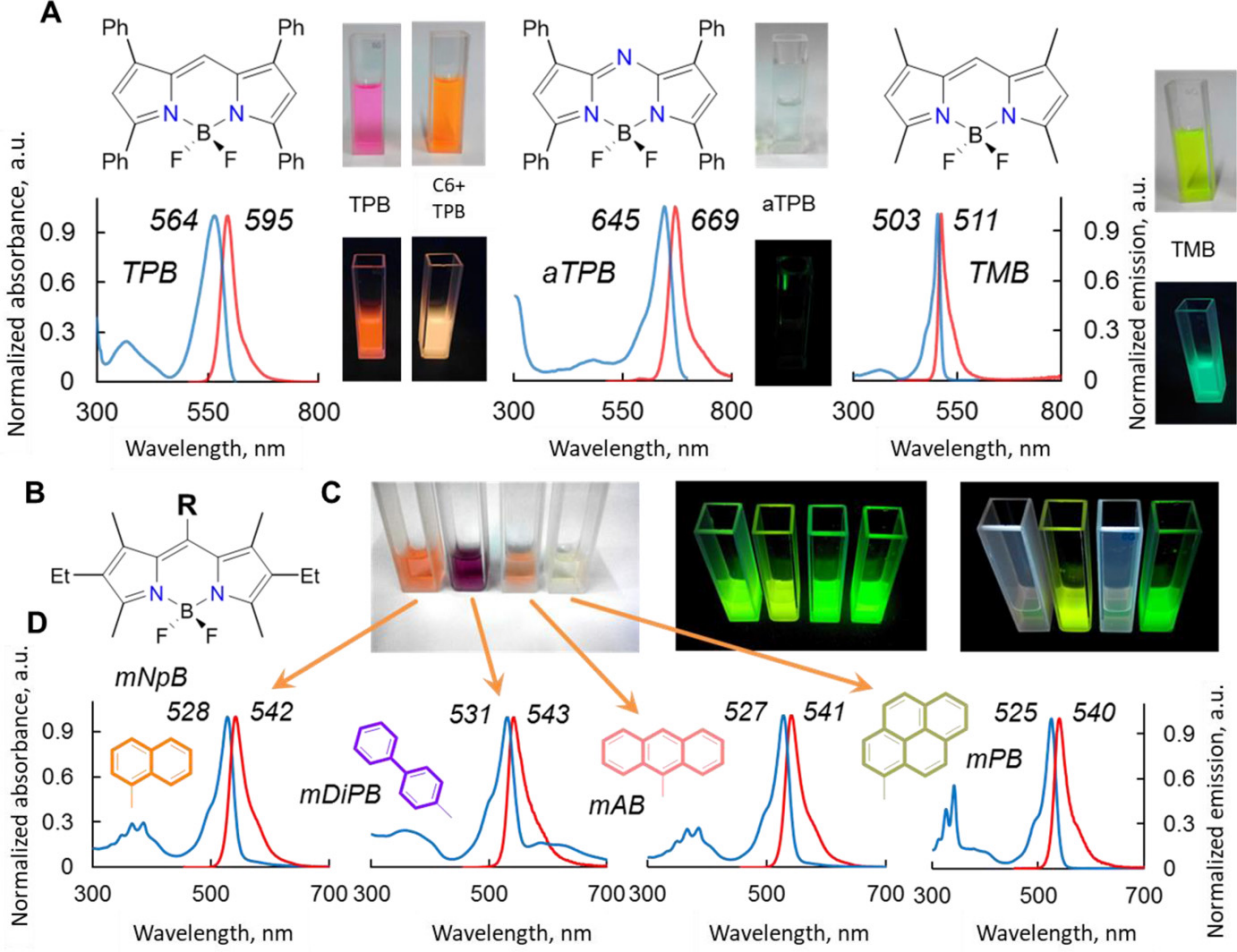
(**A**) Structural formulas of the BODIPY dyes: *tetra*-Ph-BODIPY (TPB), t*etra*-Ph-aza-BODIPY (aTPB), *tetra*-Me-BODIPY (TMB); the corresponding solutions of the dyes and their mixtures in transmitted daylight from left to right (10 µM in MeOH): TPB, the mixture of TPB/Coumarin 6 dye (C6), aTPB and TMB under daylight and under irradiation by a UV lamp at a wavelength of 365 nm, and the corresponding normalized absorption (blue) and fluorescence (red) spectra of the dyes in diluted solutions (5 µM), respectively, in MeOH. (**B**) The general structural formulas of meso-substituted BODIPYs. (**C**) The solutions of *meso*-substituted BODIPYs (10 µM in MeOH) from left to right: naphthyl-BODIPY (mNpB), diphenyl-BODIPY (mDiPB), anthryl-BODIPY (mAB), pyrenyl-BODIPY (mPB) in transmitted daylight (left), under irradiation by a UV lamp at a wavelength of 365 nm (middle) and 254 nm (right) and the corresponding normalized absorption (blue) and fluorescence (red) spectra in diluted solutions, respectively. (**D**) Normalized absorption (blue) and fluorescence (red) spectra in diluted solutions (5 µM), respectively, in MeOH. The Mendeley repository for this Fig. contains the normalized absorption and emission spectra for each dye (path: “Root – Data in Brief main dataset – Data for Fig. 2): Fig. 2 - BODIPY normalized spectra.xlsx”.

The application of BODIPY as molecular rotors is based on an interplay between the increased structural rigidity of the dye and an enhancement of the fluorescence intensity upon an increase in the dynamic viscosity of the molecular environment. Inhibition of the free rotation of the substituents, combined with the increased rigidity of the dye, leads to a reduction in non-radiative decay; hence, it enhances fluorescence by decreasing the gap distance between HOMO and LUMO. This mechanism is enabled via stabilization of the different geometries for the ground and excited states of the luminophore. Thus, in the ground state, the aromatic fragments of the BODIPY are orthogonal to each other; however, upon transition to the excited state, the conformation with coplanar fragments becomes more favorable. Here, the transition to a more stable state leads to a decrease in the LUMO energy, from which, in turn, a non-radiative transition to a higher vibrational sublevel of HOMO is possible. Thus, the decrease in the HOMO-LUMO gap and the increase in the possibility of such a transformation and transition determine the fluorescence intensity. This mechanism is known as a TICT (Twisted Intramolecular Charge Transfer)-state. The luminescent characteristics of the dyes were measured and are shown in Fig. 2.

The use of BODIPY, described here, is dictated by considering their properties, namely:

(1) High hydrophobicity;
(2) The presence of characteristic aggregation behavior affecting fluorescence;
(3) Spectral band positions located close to a therapeutic window;
(4) Structural similarity to the BPhen ligand and its Fe(II) complex;
(5) Simple synthesis scheme;
(6) Useful photophysical parameters.

The chosen dyes are known to be promising for a wide range of applications, including molecular rotors, viscosity sensors, cellular imaging agents, self-assembled systems, photoacoustic detection devices, and phototherapy.

The difference in the optical characteristics of these compounds at 254 nm UV irradiation is attributed to the substituents' rigidity. The short-wavelength irradiation is absorbed by the substituent π-system of these dyes, whereas in mDiPB and mPB the energy transfer by partial conjugation of the BODIPY core and the substituent electronic systems via rotation or vibration is also possible. In all cases, under long UV wavelengths, the light is partially absorbed directly by the dipyrrin core, which leads to the appearance of the signal in fluorescent spectra.

*3. Spectra of BODIPY dyes in a binary mixture of THF:H_2_O 5:95 (v/v)*

**Fig. 3:**
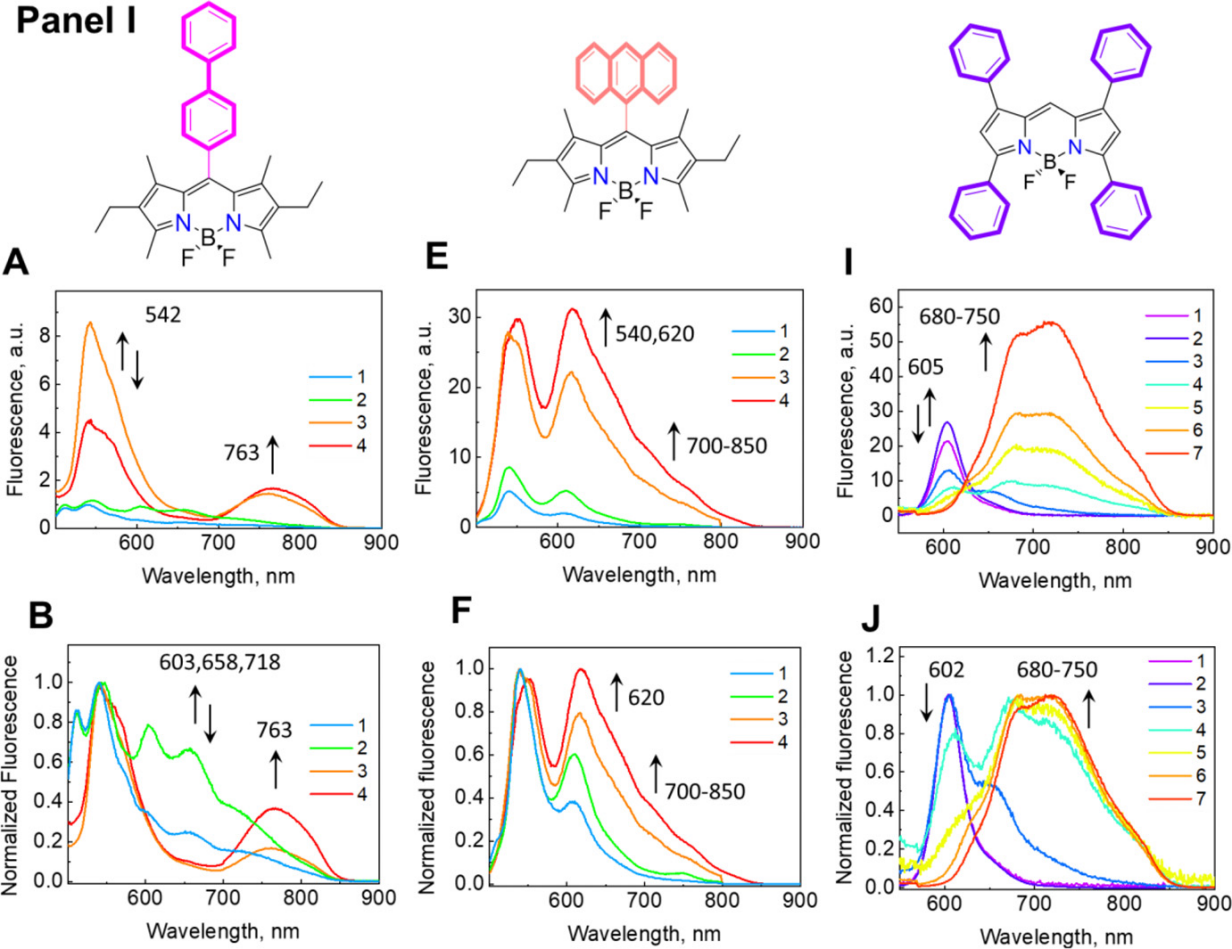

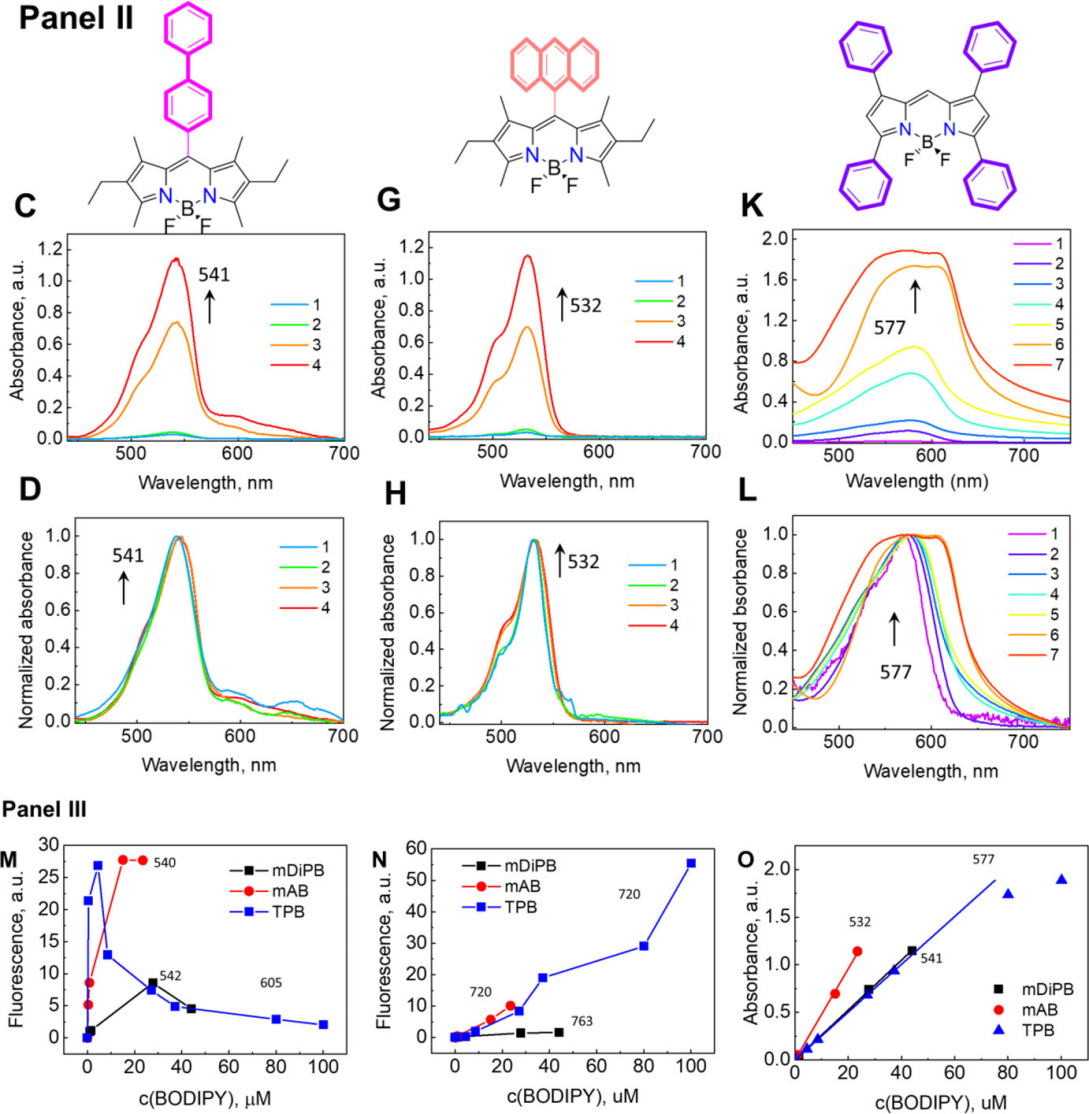
***Panel I:*** The absolute and normalized fluorescence **(A, B, E, F, J, K), *Panel II:*** The absolute and normalized absorption spectra (**C, D, G, H, L, M**) in a binary mixture of THF:H_2_O 5:95 v/v of mDiPB **(A – D)** 1 – 1.1, 2 – 1.6, 3 – 27.8, 4 – 44.1 µM; mAB **(E – H)** 1 – 0.4, 2 – 0.9, 3 – 15.1, 4 – 23.5 µM, **(I – L)** 1 – 0.5, 2 – 4.5, 3 – 8.6, 4 – 27.1, 5 – 37.2, 6 – 80.0, 7 – 100 µM. ***Panel III:*** The relative changes in fluorescence (**M, N**) absorbance (**O**) of the compounds as a function of the dye concentration. The Mendeley repository for this Fig. contains the data for plots in *.opju (Origin Pro) format (path: “Root – Data in Brief main dataset – Data for Fig. 3”).

The control measurements of the spectral parameters of the dyes were carried out in a THF:H_2_O mixture in a volume ratio of 5:95 (Fig. 3), which is the measurement method most often used to obtain BODIPY aggregates in an aqueous environment. This model is good for establishing the formation of BODIPY associates and aggregates in the presence of surfactants in MCAs and MCCs. At low concentrations (<10 µM), the investigated dyes do not form aggregates and exhibit fluorescence characteristics corresponding to their monomeric form, which are similar to the characteristics in non-polar organic solvents (e.g., MeOH, THF). An increase in the concentration of the dyes leads to changes in the fluorescence spectra, which correspond to the compound's structure. The increase in the concentration of the dye mDiPB, as well as mAB, is not accompanied by changes in their absorption spectra. Contrary to mDiPB and mAB, an increase in the TBP dye concentration led to a broadening of the adsorption band and the appearance of new peaks at 600–620 nm. These changes in the absorption spectra are attributed to the formation of the dyes’ associates. The fluorescent spectra of the mDiPB, mAB, and TBP dyes exhibited the formation of a new peak measured at >600 nm. With a further increase in the dyes’ concentration, new peaks are formed in the infrared (IR) regions; however, the peaks appeared earlier at ∼540 and 600 nm, and lost their intensity due to fluorescence quenching. This indicates the dynamic nature of the associative equilibrium, i.e., an increase in concentration leads to an increase in the number of associates.

*4. Validation of the MCC approach*

To enhance the solubility of hydrophobic compounds, except for direct chemical modification of compounds by means of introducing hydrophilic groups, the application of co-solvents, adjustment of pH, variation of the salt concentration, an increase in a specific surface, and a reduction in the particle size have been utilized. The methods of choice are based on the characteristics of compounds in relation to their prospective applications, although several approaches utilize the formation of complexes with fillers such as hydrophilic polymers and cyclodextrins.

The micellar/vesicular/liposomal approach or BODIPY encapsulation in nanotubes or organic frameworks is another powerful technique for solubilization/encapsulation to study the physicochemical properties of the obtained materials (such as aggregation or fluorescent enhancement), construction of micro-/nanoreactors, or for applications in photodynamic therapy. A hybrid, but more complicated approach combining chemical modification of a dye with organic components, allowing self-assembly into various nanostructures with spatially distinct functional regions, is applicable for developing supramolecular luminescent sensors and for anticancer treatment. Therefore, approaches that allow the development of materials with high solubilization and encapsulation capacity that protects loaded cargos without altering their optical characteristics and that provide a high fluorescence response are promising and of great importance for both biochemical and medical research.

**Fig. 4:**
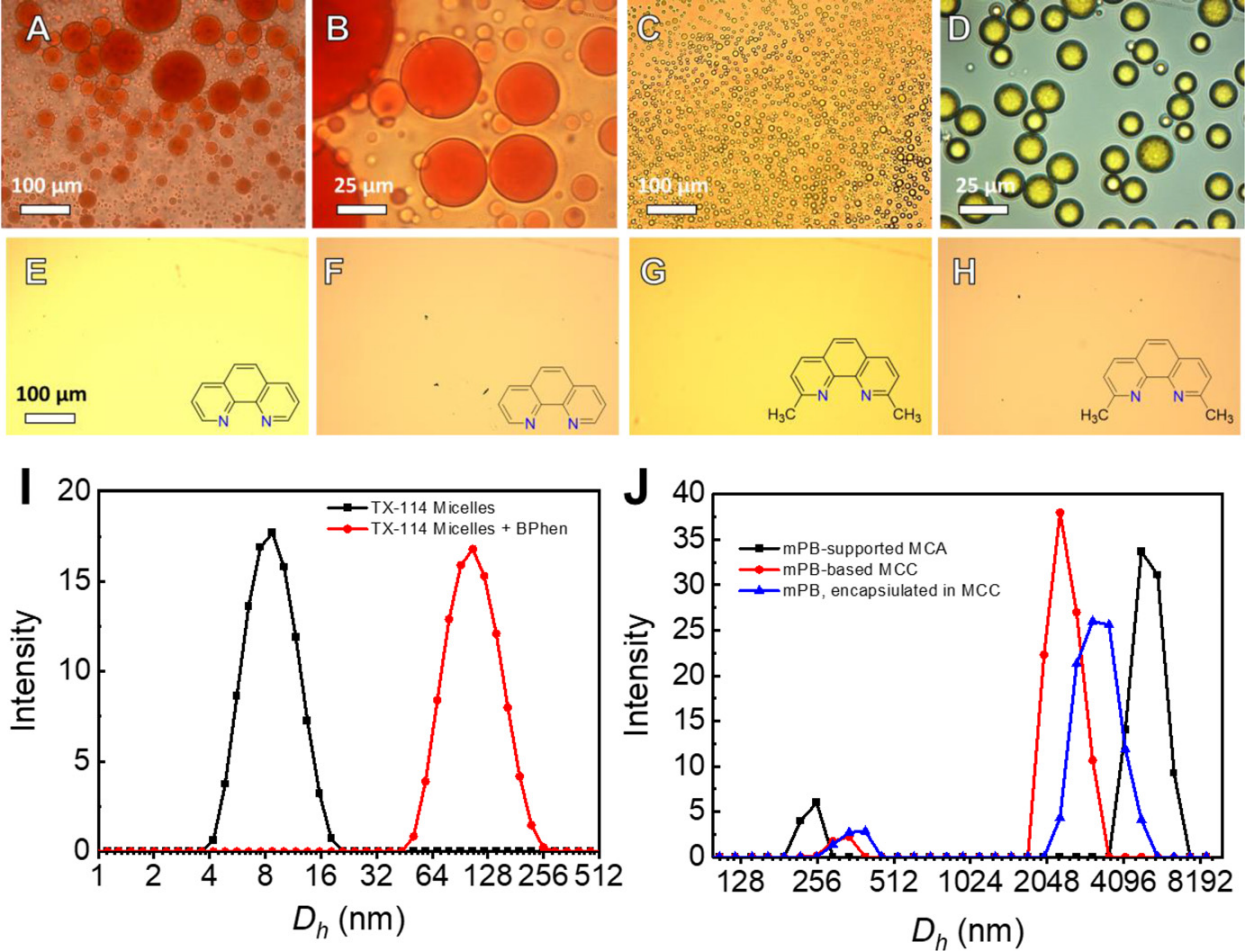
(**A, B**) Microscopy images of the micellar clusters based on Triton-114 surfactant after 40 min of incubation, prepared following the scheme in the presence of Fe^2+^ ions and (**C, D**) in the presence of Ni^2+^ ions with encapsulated Coumarin 6 fluorescent dye. The effect of BPhen chelator replacing the micelle clusterization after 30 min: onto Phen (**E, F**) and Neocuproine (**G, H**) for TX-100-based MCCs (**E, G**) and TX-114 (**F, H**) and chemical formulas of the corresponding structures. **I, J** – dynamic light scattering (DLS) spectra of TX-114 micelles, MCA, mPB-supported MCA, mPB-based MCCs, and MCC with encapsulated mPB. DLS spectra were taken after 10 min of the corresponding system formation. The Mendeley repository for this Fig. contains the data for plots in *.opju (Origin Pro) format (path: “Root – Data in Brief main dataset – Data for Fig. 4”).

Generally, in a traditional approach, the unstable hydrophobic dyes are encapsulated and thus are stabilized in surfactant micelles. These surfactant micelles may undergo interfacial stabilization, followed by clusterization, forming a MCC network; this is usually achieved by incorporating chelate ligands into a micelle interface, followed by complexation with metal ions (see the main text, Fig. 1 General approach). Traditionally established methods [7,8] for the conjugation of non-ionic surfactant micelles are based on an increase in the local surfactant concentration, which leads to a decrease in the cloud point of the surfactant and promotes phase separation. These approaches suggest that a specific hydrophobic ligand (BPhen) be incorporated into the micelle interface, forming micelle-chelator aggregates (MCAs), which are stabilized through hydrophobic interactions. The MCAs themselves are highly unstable and tend to form BPhen crystals and individual micellar phases within a short time. To stabilize the MCAs, a solution of divalent metal ions (e.g., Fe^2+^, Ni^2^) is added to bind to BPhen. An electrolyte (NaCl) is used to elevate the ionic strength and promote micellar clusterization. Introducing an electrolyte results in the formation of the M[BPhen]_3_^2+^ complex, which stabilizes the inter-micellar connections. The process of MCC formation is accompanied by the appearance of red/transparent droplets, depicted in Fig. 4, A–D; this process is regulated by the metal ions, indicating that the clusterization process has been initiated. The obtained MCCs, based on TX-100/TX-114 surfactants, have variable sizes, depending on the preparation procedure, and they can be tuned from ∼1 to ∼400 µm. The MCC formation mechanism is specific and requires the presence of all components; the absence of one of the components in the mixture causes the MCC formation to fail.

The formed features, stability, kinetics, and the optimal formation conditions (component concentrations and their ratios; metal ions and solvent replacement) of the TX-100/TX-114-based MCCs, as well as the effect of external factors (e.g., pH, temperature, and the addition of competing chelators) on the clusterization process, system stability, and the encapsulation abilities using Coumarin 6 have been studied and reported previously (Fig. 4, C, D) [9,10]. Despite their structural similarity, TX-114 has a lower cloud point temperature, compared with TX-100; therefore, the effect of a cloud point change on the micellar clusterization process was studied to investigate BODIPY encapsulation and the fluorescent characteristics.

An attempt to replace hydrophobic BPhen with more hydrophilic Phen or neocuprione failed (Fig. 4, E–H). Even after 5 days of incubation, both ligands did not induce any clustering.

According to the DLS spectra (Fig. 4, I, J), a pure TX-114 surfactant (Table 1) solution of 3–11 mM initially contains stable micelles with a size of 4–16 nm. After the addition of BPhen, however, the major peak shifted to ca. a 100 nm region, testifying to the formation of unstable MCA. Introducing mPB to MCA for its stabilization resulted in the formation of a new peak of ca. 5–7 µM after 10 min, along with the gradual disappearance of a peak of MCA at 100–200 nm. The same phenomenon is observed during the encapsulation of mPB at MCCs. The addition of mPB after 10 min also leads to the formation of a peak >1 µM due to the formation of mPB-based MCCs.

**Table 1:** The structural formula of Triton-X surfactants and their properties (according to the Sigma-Aldrich specification).


Surfactant	*n* (chain length)	CMC, mM (20–25°C)	Cloud point/pour point,°C	Hydrophobic-lipophilicbalance
TX-100	9–10	0.2-0.9	65 / 7	13.5
TX-114	7–8	0.2	23 / 9	12.4

After the micellar conjugation concept was proposed, it was suggested and shown that not only BPhen family-based ligands could be used for the micellar conjugation process—several designed Phenanthroline molecules could also be used for this purpose. Later, it was hypothesized that some molecules of other classes that are structurally similar to the BPhen ligand and their respective metal complexes with similar properties but possessing different features may be used for this purpose as well. This hypothesis is based on the fact that a chelator (and its metal complex, correspondingly) rigidity serves as a means for promoting *π-π* interactions and for preserving metal binding affinity, regardless of the surfactant environment; it appears to be a mandatory property in micelle conjugation.

In this regard, our study also focused on the formation of micellar coordination clusters (MCCs) composed of non-ionic surfactants of the Triton-X family, and related fluorescent dyes that are structurally similar to the BPhen ligand and its related metal complex (BODIPY). The advantage of the proposed stabilization approaches lies in the absence of precipitants, their high ionic strength, and temperature alterations. We designed and developed alternative routes for dye solubilization and stabilization; their efficiency and applicability have been compared to the traditional approach. We investigated whether BODIPY dyes can act as “supporting molecules” for micelle-chelator aggregates (see the main text, Fig. 1, approach II). Then, we examined the ability of the dyes to self-stabilize via micellar cluster formation (see the main text, Fig. 1, approach III). Finally, we investigated the ability of the MCC BODIPY assemblies to sense hydrophilic protein molecules. Moreover, since the BODIPY dyes make MCC fluorescent, it is possible to track any changes in their inner structure upon their interaction with other molecules of interest, e.g., membrane proteins or bio-objects.

In this regard, in addition to the visual manifestation of micellar cluster formation, we present some information about the structure of micelles, their size, and BODIPY molecules, as well as a hypothesis about the placement of BODIPY in micellar space.

**Fig. 5:**
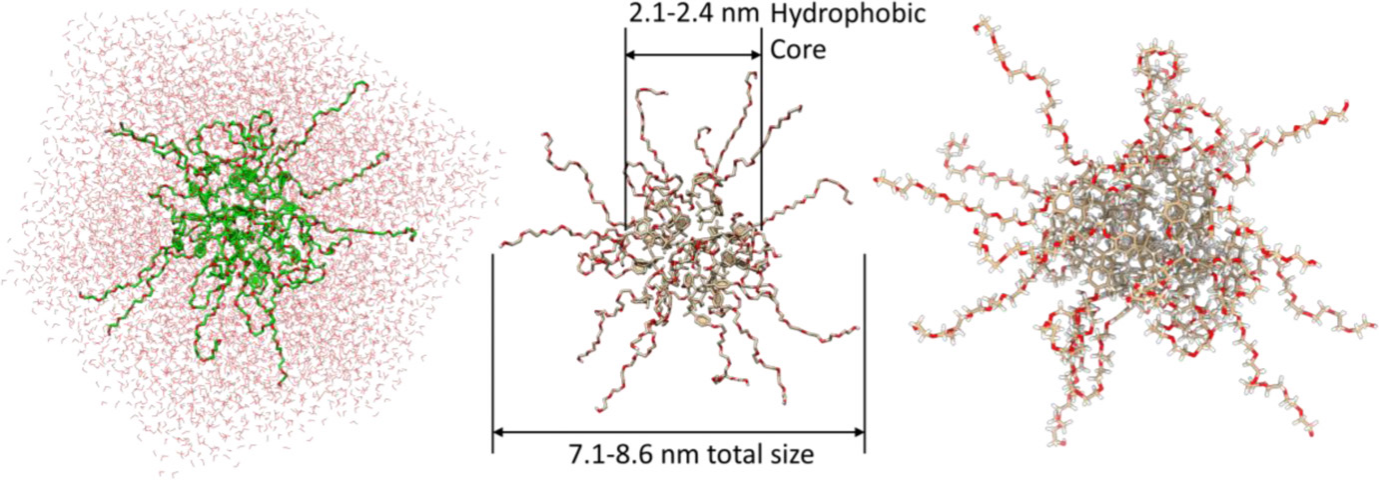
TX-114 micelle model (25 TX-114 molecules, spherical approximation) solvated in a water cube of a ca. 6 × 6 × 6 nm side (right) and in an isolated state: (center) the water molecules removed, without backbone hydrogens and (right) with them calculated using CHARMM. The Mendeley repository for this Fig. contains the input/output data files obtained from CHARMM-GUI [6] (path: “Root – Data in Brief main dataset – Micelle models – CHARMM), as well as video files of the micelle rotation in a naked and solvated state generated from ChimeraX using the data from CHARMM (path: “Root – Data in Brief main dataset – Micelle models – Media”).

According to the molecular modelling calculations, performed in CHARMM, in the simple case, TX-114 micelle (Fig. 5) represents a spherical agglomerate of surfactant molecules with a size up to ca. 9 nm in diameter (calculated for 25 TX-114 molecules), which is in accordance with the DLS experiments. Under other conditions, the assembly type might be different and the micelle might become cylindrical, disk-, or thread-like. These more complicated cases might be realized under elevated surfactant concentrations, elevated temperature, or upon increasing the ionic strength of a solution and are intended to be a part of a separate publication. For considering the assembly, the estimated diameter of the hydrophobic core is ca. 2.1–2.4 nm.

**Fig. 6:**
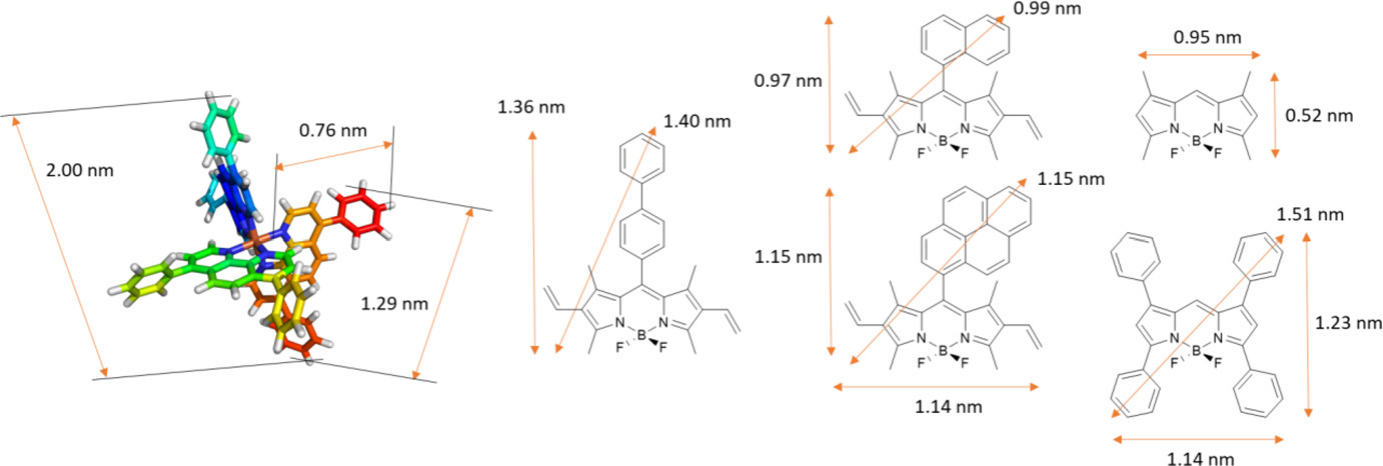
Estimated size of the Fe-BPhen complex (left) and several major BODIPY structures (right) with drawn dimensionalities. To determine the distances, all the structures were optimized with Gaussian G09 software using the DFT/B3LYP/6-311G (d,p) method. Molecule visualization – Chimera 1.16, structural formulas – ChemDraw 2016.

Furthermore, the structures of Fe[BPhen]_3_ and a set of BODIPY structures have been built and their geometry has been quantum-chemically optimized to a minimum of energy (Gaussian G09 software, DFT/B3LYP/6-311G (d,p) method, in vacuum, Fig. 6).

The major concept of micellar conjugation assumes that the hydrophobic part of the BPhen during micelle interaction and MCA formation is located inside of the hydrophobic core, whereas the nitrogen atoms are available for complexation with Fe(II) to form the Fe[BPhen]_3_ complex. According to these calculations, the maximum length of the BPhen ligand is ca. 0.76 nm, its length is ca. 1.29 nm, and this part sits entirely inside of the hydrophobic core of the micelle. Considering the octahedral configuration of the Fe ion, the maximum length of the Fe-BPhen complex is ca. 2 nm in all three dimensions. Therefore, the distance between the two opposite nitrogen atoms taking part in bond formation with the Fe^2+^ ion is ca. 0.48 nm (2–0.76*2); this may correspond to the shortest intermicellar distance with respect to the interactions between hydrophobic shells (not center-to-center). It is also known that replacement of BPhen onto a less hydrophobic and smaller Phen ligand does not lead to micelle conjugation. In addition, BPhen itself forms pre-micellar clusters (also called MCA); however, it cannot stabilize it for a long enough time to form MCCs and consequently, it separates into a crystalline phase. In addition, the presence of two phenolic rings in a BPhen structure that provides molecular rotor properties to this molecule, which is similar to the BODIPYs investigated, is not enough to form MCCs due to its specific structure, which allows the BPhen ligand to bind preferentially only to a single micelle. As was pointed out in the main text, TMB also cannot be used for MCC formation. Therefore, the size of the molecule that can be used for MCC formation should not be less than the size of BPhen, and it has to have an extended pi-electronic system. These criteria meet all of the investigated BODIPYs except for TMB; mNpB is considered as a "border compound". Based on these assumptions, the penetration depth of several compounds can be calculated. Taking the maximum distance for all of the compounds, and assuming that, on average, half of the molecule may interact with one micelle, the penetration depth for mNpB is ca. 0.26–0.50 nm, for mPB it is ca. 0.34–0.58 nm, for DiPB it is ca. 0.46–0.70 nm, and for TPB it is ca. 0.52–0.76 nm. These assumptions are based on the hypothesis that only a single molecule of BODIPY takes part in the intermicellar interactions. If it is assumed that BODIPY may form aggregates (*J, H-,* or any other tile-based aggregates), these distances may vary as well if the micelle size/type changes.

*5. TX-100-based MCCs*

**Fig. 7:**
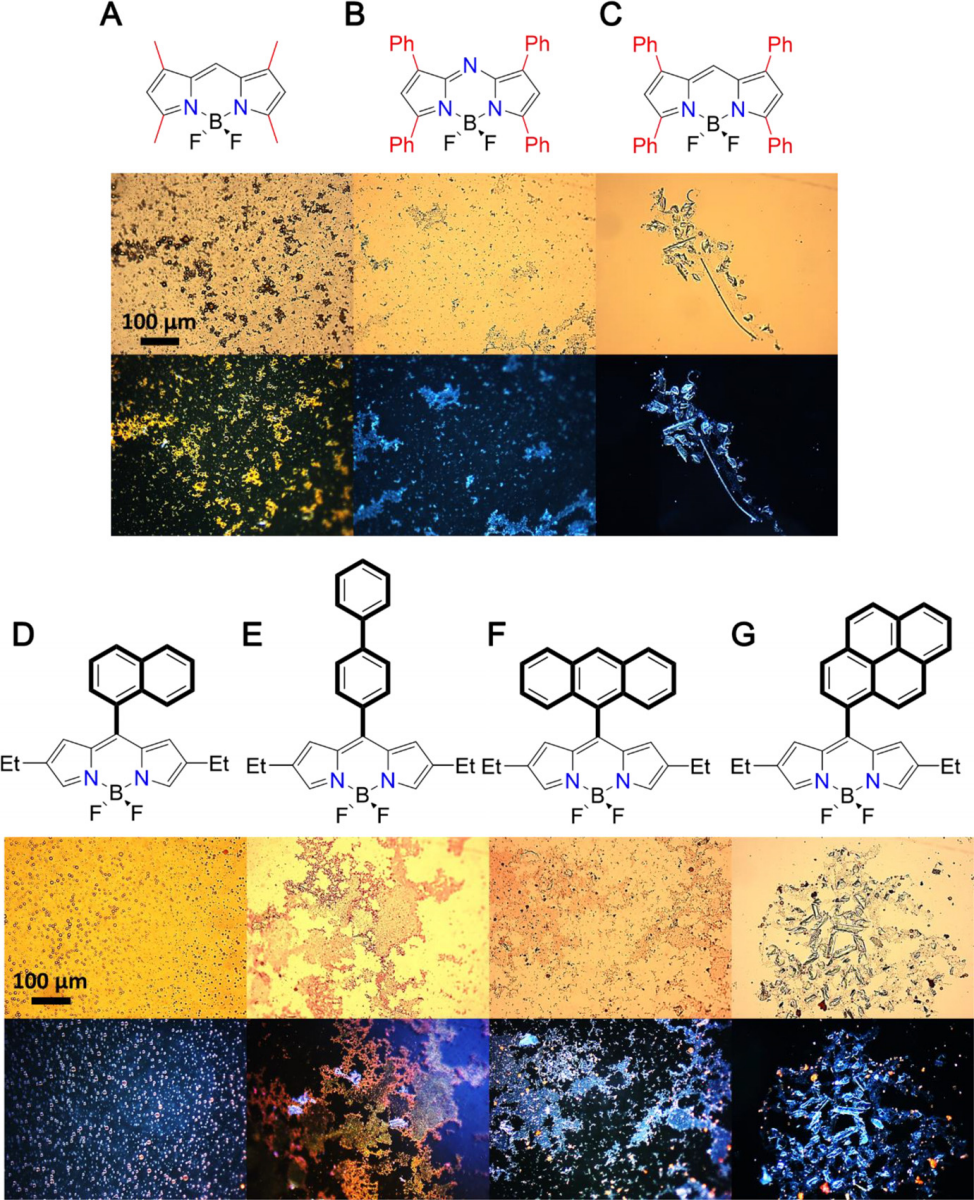
Optical microscope images in transmitted light (top panel) and the corresponding images in the dark-field regime (bottom panel) during the first hour of the formation of BODIPYs in TX-100-based MCCs (**A–G**). The chemical structures of the dyes are shown on the top of the microscopy images.

The micellar clusterization approach has been tested for the TX-100 surfactant application; however, the initial concentration of the dyes should be reduced to prevent undesired crystallization (Fig. 7).

*6. CLSM images of BODIPY-encapsulated MCCs*

**Fig. 8:**
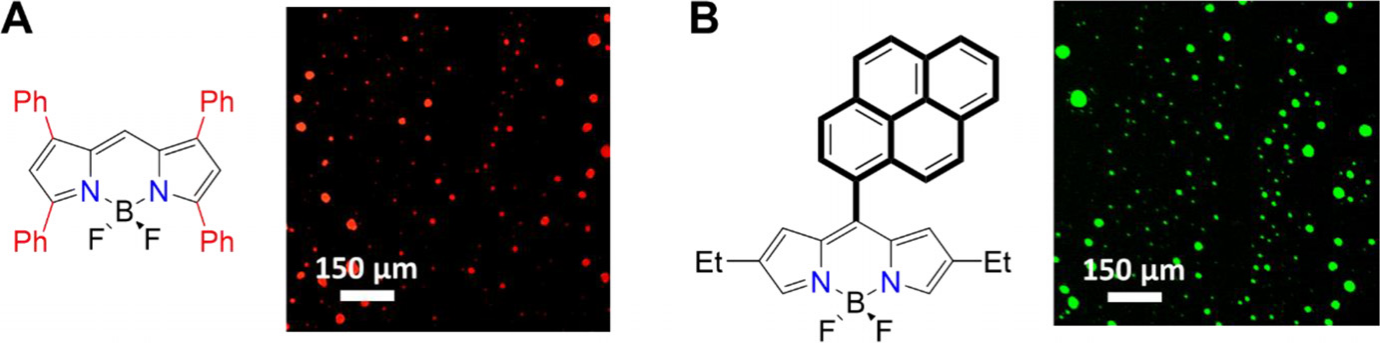
CLSM images of TPB (λ_ex_ = 547 nm, **A**) and mPB (λ_ex_ = 488 nm, **B**) in MCCs during the first hour of formation (see the main text in Fig. 1, approach 1 and Fig. 2). Scale bars are 150 µM.

Encapsulated dyes in TX-114-based MCCs preserve their intrinsic fluorescence (Fig. 8).

**Fig. 9:**
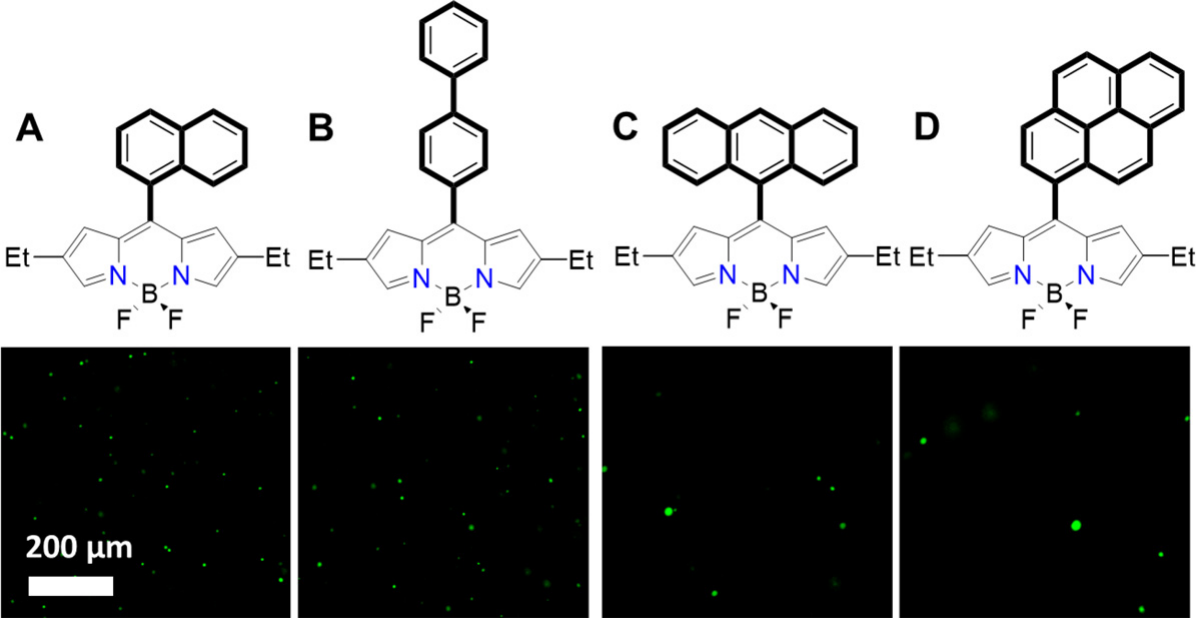
CLSM images (λ_ex_ = 488 nm, insets) for (**A**) mNpB, (**B**) mDiPB, (**C**) mAB, and (**D**) mPB used as hydrophobic molecular support for the MCA after 10 min of formation (see the main text in Fig. 1, approach 2 and Fig. 4). Scale bars are 200 µM.

*7. CLSM images of BODIPY-supported MCAs*

BODIPY-supported TX-114-based MCAs also possess strong fluorescence (Fig. 7).

*8. Spectra of BODIPYs titrated with TX-114*

**Fig. 10:**
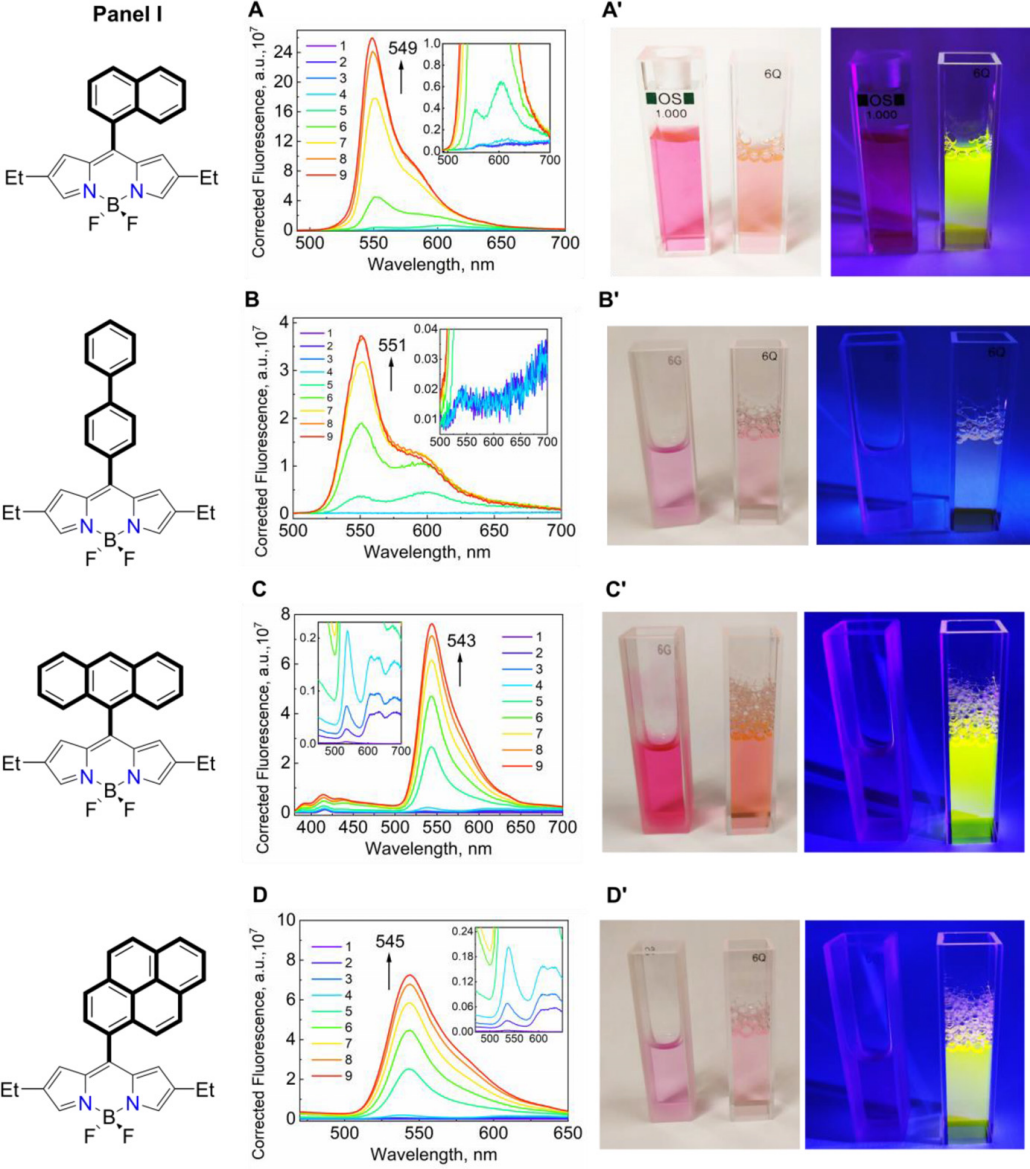

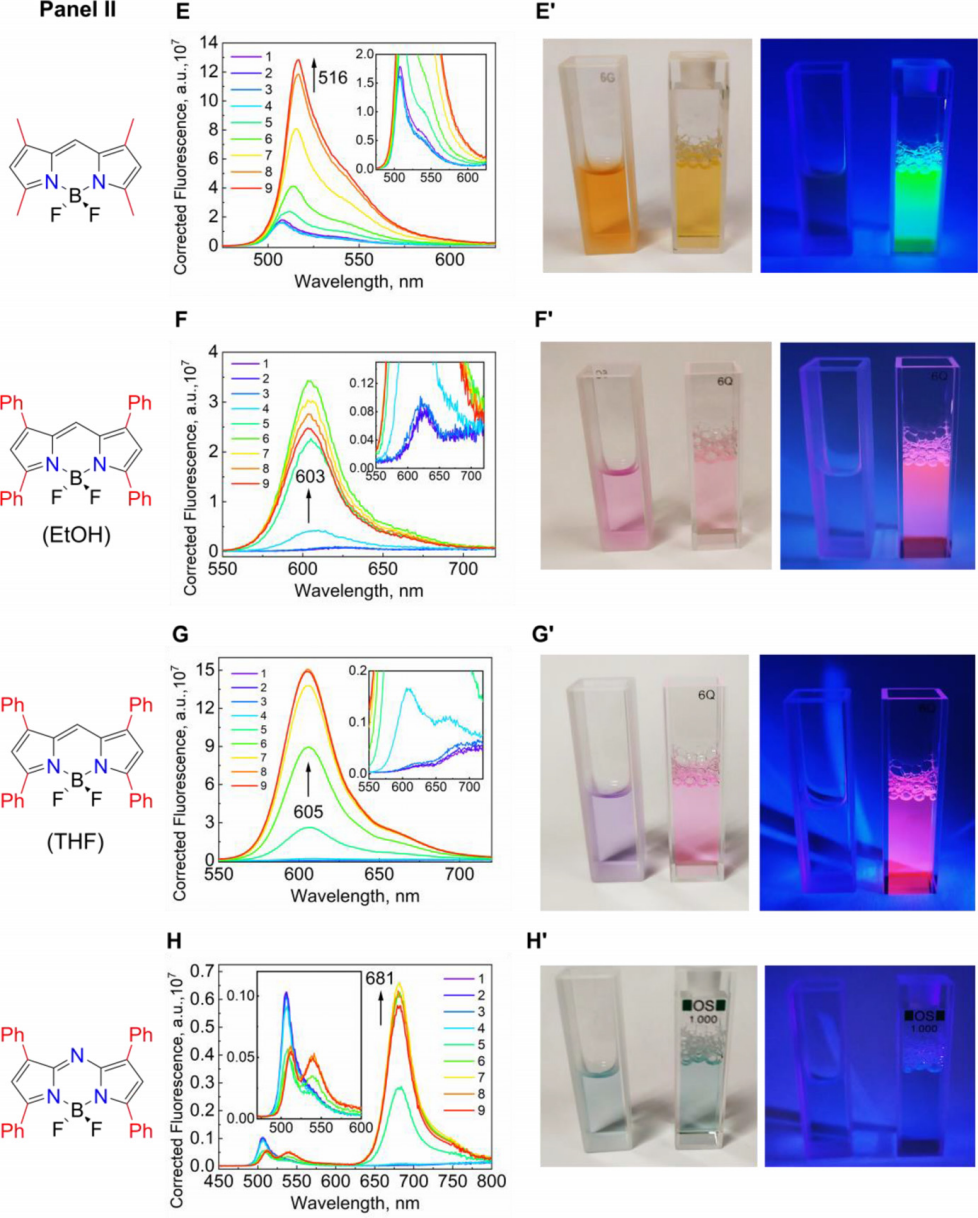
***Panel I*:** Changes in the fluorescence spectra of (**A**) mNpB, (**B**) mDiPB, (**C**) mAB, and (**D**) mPB; ***Panel II*:** Changes in the fluorescence spectra of (**E**) TMB, (**F**) TPB (solvent – EtOH), (**G**) TPB (solvent – THF), and (**H**) aTPB during the titration by TX-114; the final concentrations of TX-114 in solution are (in mM): 1 – 0, 2 – 0.05, 3 – 0.1, 4 – 0.2, 5 – 0.5, 6 – 1, 7 – 2, 8 – 3, and 9 – 4, and the corresponding changes in the dye colors (**A' – H')** for their aqueous solutions in the absence (left cuvette) and in the presence of 4 mM (right cuvette) of TX-114 under daylight illumination (left image) and under irradiation of 365 nm UV light (right image). The Mendeley repository for this Fig. contains the data for plots in *.opju (Origin Pro) format (path: “Root – Data in Brief main dataset – Data for Fig. 10”).

**Fig. 11:**
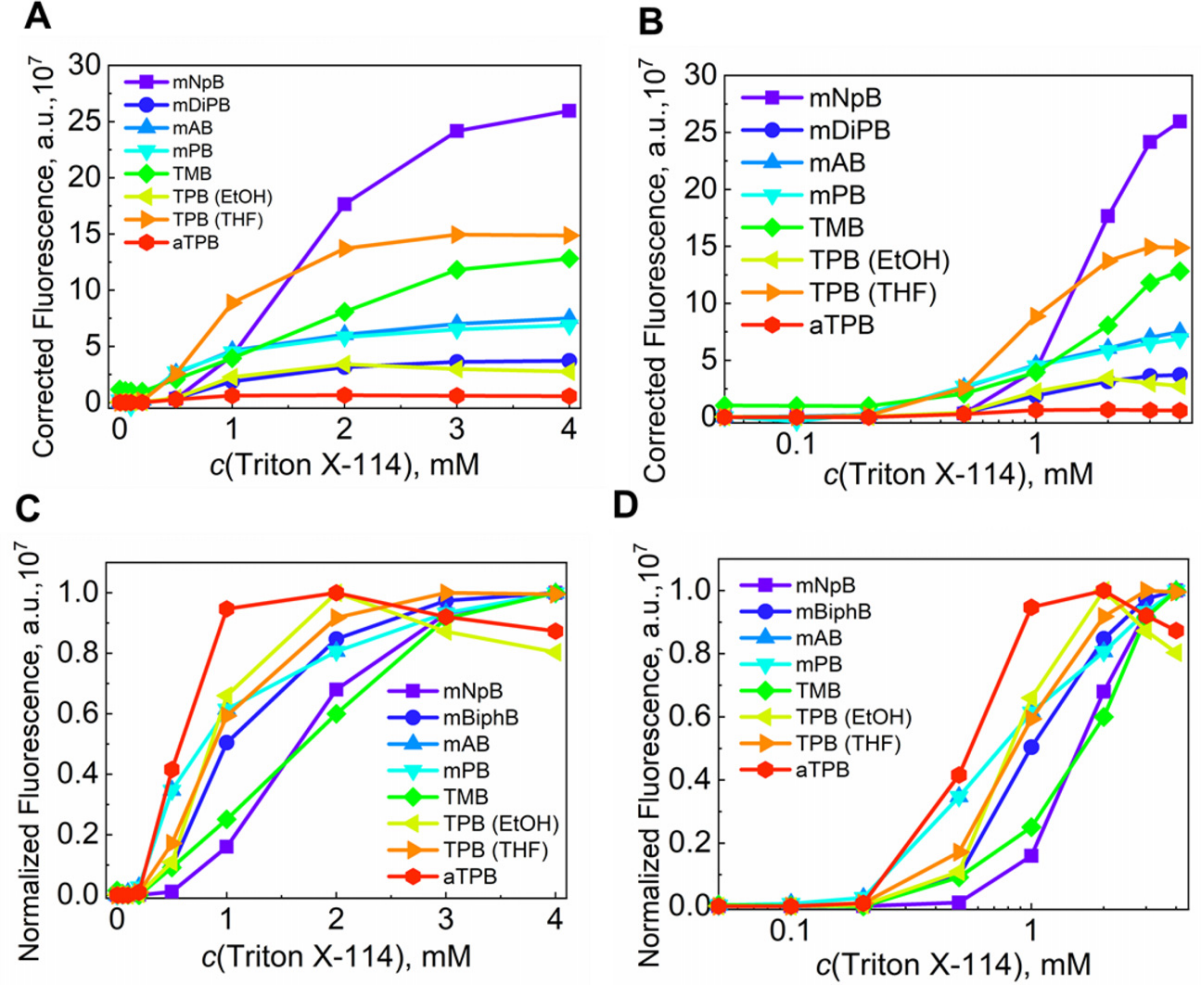
(**A, B**) The changes in the absolute and (**C, D**) normalized fluorescence at peak maximum for the dyes in the presence of TX-114 in normal (**A, C**) and a semi-log scale on the X-axis (**B, D)**. The Mendeley repository for this Fig. contains the data for plots in *.opju (Origin Pro) format (path: “Root – Data in Brief main dataset – Data for Fig. 11”).

The titration series have been carried out for all of the dyes to establish their fluorescence behavior (Figs. 10, 11, and Table 2).

**Table 2:** Titration of TX-114 into BODIPYs.

*c^0^ (BODIPY)* µM	*V^0^ (BODIPY)* *µL*	*V^0^ (H_2_O) mL*	*V^add^ (TX-114, 11 mM)* *µL*	*V^total^ mL*	*c(TX-114) mM*	*c(BODIPY)* µM
40	40	1.453	0	1.493	0	1.07
			6.818	1.500	0.05	1.07
			6.910	1.507	0.1	1.06
			13.9	1.521	0.2	1.05
			43.5	1.564	0.5	1.02
			79	1.643	1.0	0.97
			182	1.825	2.0	0.88
			227	2.052	3.0	0.78
			295	2.347	4.0	0.68

*9. Changes in the fluorescence spectra of the dyes in TX-114, MCAs, and MCCs*

**Fig. 12:**
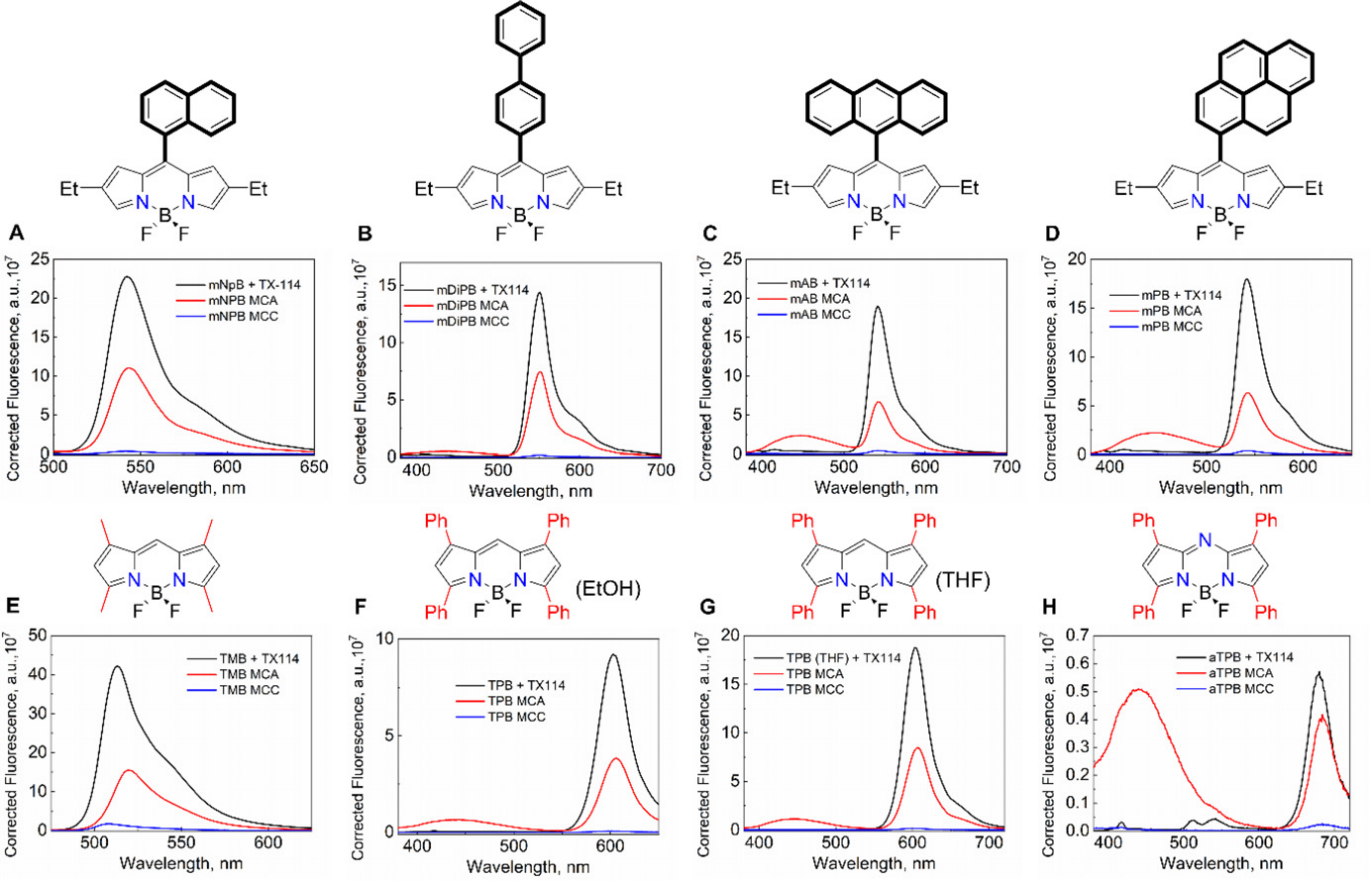
Changes in the fluorescence spectra of mNpB (**A**), mDiPB (**B**), mAB (**C**), mPB (**D**), TMB (**E**), TPB (solvent – EtOH, **F**), TPB (solvent – THF, **G**), and aTPB (**H**) in TX-114 (1.35 mM, black), after the addition of BPhen (MCA, red) and after the addition of a salt solution (MCCs, blue). The Mendeley repository for this Fig. contains the data for plots in *.opju (Origin Pro) format (path: “Root – Data in Brief main dataset – Data for Fig. 12”).

The fluorescence for all of the dyes has been measured and compared in all of the self-assembled surfactant systems (Fig. 12).

*10. Kinetic studies for mAB, mPB, and TPB fluorescence during MCA formation*

**Fig. 13:**
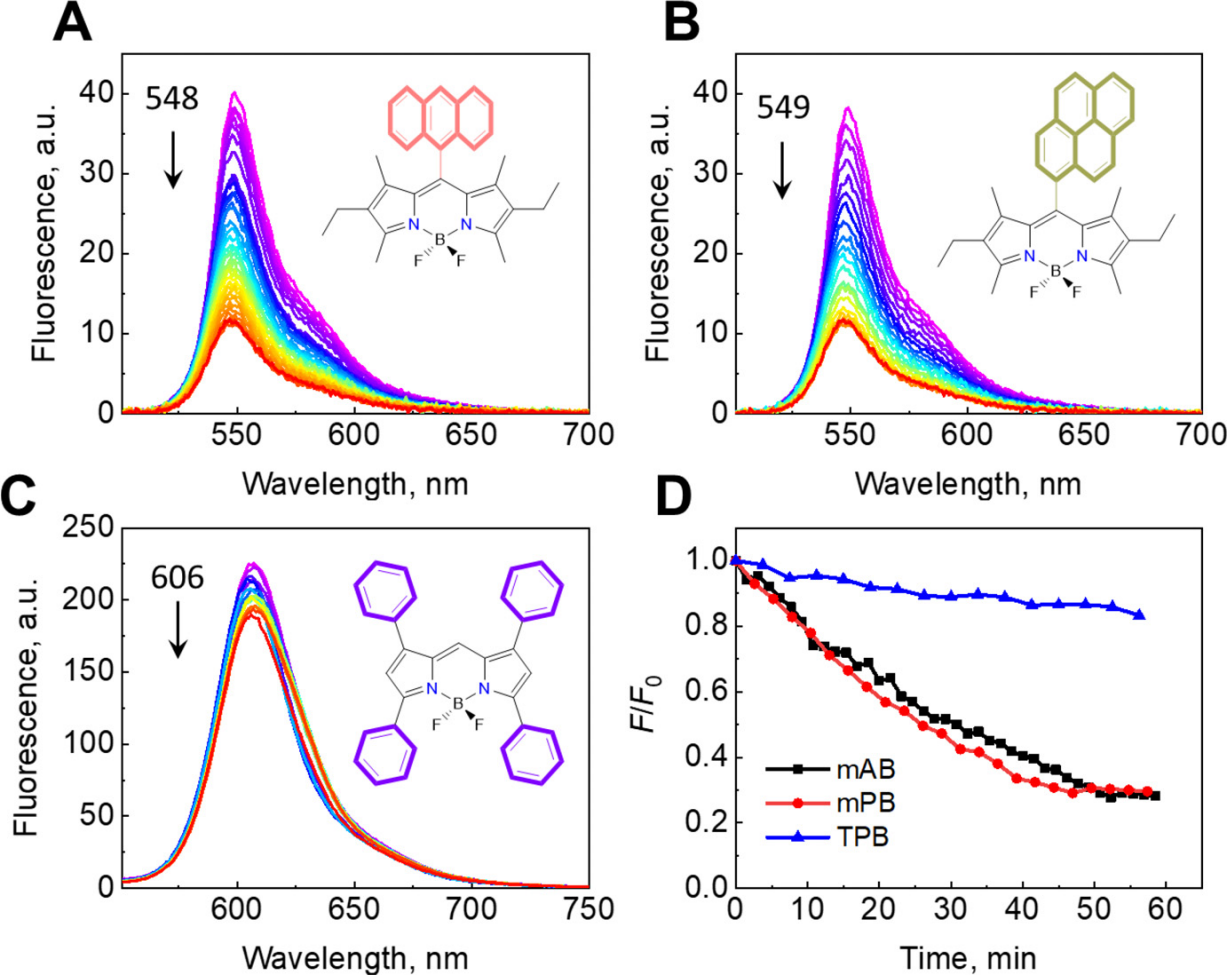
Changes in the fluorescence spectra of MCAs supported by mAB (**A**), mPB (**B**), and TPB (**C**), and the relative changes in the fluorescence maxima for the investigated systems (**D**). The Mendeley repository for this Fig. contains the data for plots in *.opju (Origin Pro) format (path: “Root – Data in Brief main dataset – Data for Fig. 13”).

Kinetic studies of the fluorescence quenching have been carried out for *mAB, mPB, and TPB during MCA formation (*Fig. 13*).*

*11. Interaction of the meso-BODIPY dyes with BSA protein*

**Fig. 14:**
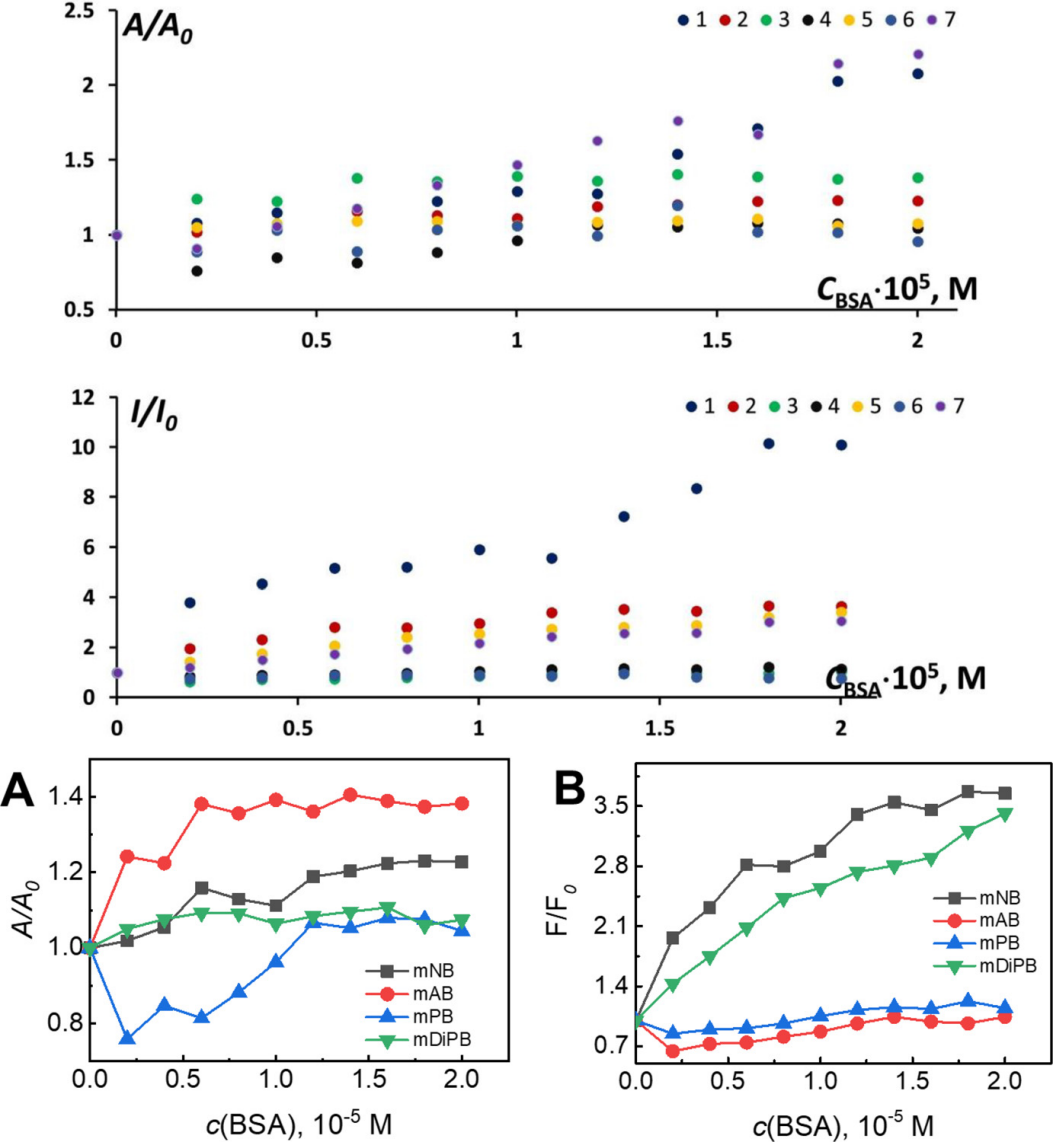
**Top panel:** Relative intensities of absorption and emission at the maxima measured upon BSA concentration variation for mNB (2), mAB (3), mPB (4), and mDiPB (5), reproduced from [11]. Copyright 2018, Elsevier; **Bottom panel:** Detailed changes in the relative intensities of (**A**) absorption and (**B**) the fluorescence maxima of the mNB, mAB, mPB, and mDiPB at different BSA concentrations, adapted from [11]. Copyright 2018, Elsevier.

Previous studies on the BSA and *meso*-substituted dyes showed that for certain BODIPYs their fluorescence may increase (Fig. 14) as well as their aggregative state.

BSA has the highest binding constants for TX-114 [12]. The thermal analysis revealed binding constants of log(K_1_K_2_) = 8.10 (TX-114), 7.87 (TX-100), which indicates the existence of two cooperative sites and 15 thermodynamically identical sites. The conformational changes in protein were observed only when the surfactant concentration reached the CMC point [12].

*12. Structure and description of the data in the Mendeley repository.*

Root // Data in Brief main dataset //

// Data for BODIPY synthesis (Section 1. Meso- and tetra-substituted BODIPY synthesis)

**Table:** 

1H-NMR.xlsx Elemental analysis.xlsx MALDI-TOF.xlsx UV-VIS Spectra.xlsx	Data for 1H-NMR, elemental analysis, MALDI-TOF, and UV-Vis spectra data for the synthesized BODIPYs

// Figures

**Table:** 

Scheme 1.png Scheme 2.png Fig. 2.png Fig. 3.png Fig. 4.png Fig. 5.png Fig. 6.png Fig. 7.png Fig. 8.png Fig. 9.png Fig. 10.png Fig. 11.png Fig. 12.png Fig. 13.png	Set of all the schemes and figures (as well as microscopy images) presented in the current paper

// Tables

**Table:** 

Table 1 - Surfactant characteristics.xlsx Table 2 - Titration data.xlsx	Table showing the Triton-X family surfactants’ basic characteristics and an explanation table for the titration data of the studied BODIPY dyes by TX-114 solution

// Data for Fig. 2

**Table:** 

Fig. 2 - BODIPY normalized spectra.xlsx	Data for normalized absorption and fluorescence spectra of diluted solutions of the studied BODIPY dyes in MeOH

// Data for Fig. 3

**Table:** 

Fig. 3A mDiPB Fluorescence.opju Fig. 3B mDiPB Normalized Fluorescence.opju Fig. 3C mDiPB Absorbance.opju Fig. 3D mDiPB Normalized Absorbance.opju Fig. 3E mAB Fluorescence.opju Fig. 3F mAB Normalized Fluorescence.opju Fig. 3G mAB Absorbance.opju Fig. 3H mAB Normalized Absorbance.opju Fig. 3I TPB Fluorescence.opju Fig. 3J TPB Normalized Fluorescence.opju Fig. 3K TPB Absorbance.opju Fig. 3L TPB Normalized Absorbance.opju Fig. 3M Fluorescence spectra maximum 1 vs concentration.opju Fig. 3N Fluorescence spectra maximum 2 vs concentration.opju Fig. 3O Absorption spectra maximum 1 vs concentration.opju	Data for absolute and normalized fluorescence and the absorbance spectra of the studied BODIPY dyes in a binary mixture of THF:H_2_O 5:95 v/v and the relative changes in fluorescence absorbance of the compounds as a function of the dye concentration

// Data for Fig. 4

**Table:** 

Fig. 3I DLS_Micelles, + BPhen.opju Fig. 4J DLS_Kinetics.opju	Data for dynamic light-scattering spectra of TX-114 micelles, MCA, mPB-supported MCA, mPB-based MCCs, and MCC with encapsulated mPB

// Micelle models (for Fig. 5)

**Table:** 

- CHARMM Input tables.docx Output file after Step 3.tgz Output file after Steps 4-1.tgz Output file after Step 4.tgz Output file after Step 5.tgz Output file after Step 6.tgz - Media Naked micelle.mp4 Solvated micelle.mp4	Data for the TX-114 micelle model (25 TX-114 molecules, spherical approximation) solvated in a water cube of ca. 6 × 6 × 6 nm. CHARMM-GUI input/output files (*.tgz archives) after every step of simulation and video files *.mp4 demonstrating naked and solvated micelle rotation over Z-axis visualized with CHIMERA-X software

// Data for Fig. 10

**Table:** 

Fig. 10A mNpB.opju Fig. 10B mDiPhB.opju Fig. 10C mAB.opju Fig. 10D mPB.opju Fig. 10E TMB.opju Fig. 10F TPB (EtOH).opju Fig. 10G TBP (THF).opju Fig. 10H aTBP.opju	Data for the changes in the fluorescence spectra of the studied BODIPY dyes during the titration by TX-114

// Data for Fig. 11

**Table:** 

Fig. 11 - Corrected-Normalized fluorescence.opju	Data for the changes in the absolute and normalized fluorescence at peak maximum for the dyes in the presence of TX-114 in normal and a semi-log scale on the X-axis

// Data for Fig. 12

**Table:** 

Fig. 12A mNpB TX114 MCC MCA.opju Fig. 12B mDiPB TX114 MCC MCA.opju Fig. 12C mAB TX114 MCC MCA.opju Fig. 12D mPB TX114 MCC MCA.opju Fig. 12E TMB TX114 MCC MCA.opju Fig. 12F TPB (EtOH) TX114 MCC MCA.opju Fig. 12G TPB (THF) TX114 MCC MCA.opju Fig. 12H aTPB TX114 MCC MCA.opju	Data for the changes in the fluorescence spectra of the studied BODIPYs after the addition of BPhen and after the addition of a salt solution

// Data for Fig. 13

**Table:** 

Fig. 13A mAB kinetics spectra.opju Fig. 13B mPB kinetics spectra.opju Fig. 13C TPB kinetics spectra.opju Fig. 13D mAB, mPB, TPB kinetics.opju	Data for changes in the fluorescence spectra of MCAs supported by mAB, mPB, and TPB, and the relative changes in the fluorescence maxima for the investigated systems

## 2 Experimental Design, Materials and Methods

### 2.1 Materials

Non-ionic surfactants Polyethylene glycol tert-octylphenyl ethers, Triton TX-100 (M_avg_ = 625 g/mol, for molecular biology) and TX-114 (M_avg_ = 537 g/mol, for molecular biology); 4,7-diphenyl-1,10-phenanthroline (bathophenantroline, BPhen, ≥ 99%), 1,10-phenanthroline (Phen, ≥ 99%), 2,9-Dimethyl-1,10-phenanthroline (neocuproine, DMPhen, ≥99.0%); NaCl (BioXtra, ≥ 99.5%); FeSO_4_ (heptahydrate, ACS reagent, ≥ 99%), NiSO_4_ (anhydrous, 98%); Coumarin 6 (≥ 99%); MeOH (anhydrous, 99.8%), and tetrahydrofuran (THF, anhydrous, 99.9%) were obtained from "Sigma-Aldrich" (USA). Triple distilled deionized metal-free water (TDW) was used in all experiments. Specific materials for BODIPY synthesis are described in Section 1.

### 2.2 The Procedure for Micellar Cluster Preparation

The main process refers to the procedure described in the main paper [1]. Briefly, 45 µL 3 mM TX-100/TX-114 surfactant (in an Eppendorf test tube) was mixed with 5 µL of a freshly prepared 20 mM BPhen (Phen or DMPhen) in MeOH and mixed by using a vortex up to 5 s. Next, 50 µL of a solution containing 56 µL of TDW, 40 µL 1 M NaCl, and 4 µL 100 mM FeSO_4_ or NiSO_4_ was added to obtain 100 µL of a solution. Finally, aliquots (5 µL) of the resulting solution were dropped immediately after their preparation on siliconized cover slides (22 × 22 mm, Hampton Research) or Menzel-Glaser glass (18 × 18 mm). The drops were incubated at 18–20 °C in a silicon grease-sealed reservoir (24-well tissue culture plate VDX (Hampton Research) or Corning, Inc.) containing 0.5 ml 200 mM NaCl or H_2_O.

### 2.3 Procedures For Dye Encapsulation in MCC, Dye Support of MCA, and Dye-Based MCC Preparation

Dye encapsulation in MCC, dye support of MCA, and dye-based MCC preparation have been carried out via one of two equivalent routes that can be scaled up. Briefly, 1 µL of a saturated solution of a dye (ca. 1 mM) in MeOH was added to 9 µL of freshly prepared standard MCCs prepared as described above (up to 5 min after adding the salt solution to MCAs) on a coverslip, followed by gentle aspiration. According to the second route, 12.5 µL of a saturated solution of a dye (ca. 1 mM) was added to 50 µL of a freshly prepared MCA solution (45 µL 3 mM TX-100/TX-114 mixed with 5 µL of 20 mM BPhen in MeOH) under vigorous stirring, followed by the addition of 50 µL of salt solution (56 µL of TDW, 40 µL 1 M NaCl, and 4 µL 100 mM NiSO_4_). In case of dye support of MCA, the step of salt solution addition is skipped. Finally, 10 µL of the obtained mixture was transferred to a coverslip.

#### 2.3.1 Spectroscopy

Electronic absorption spectra were recorded using Agilent-Varian Cary 100 (USA), Aquilon SF-104 (Russian Federation), and SOLAR SM2203 (Belarus) spectrophotometers in a 1 × 1 cm quartz cuvette. UV–Vis spectra were recorded in the range of 190–1100 nm with measurement accuracies of ± 0.03 on the optical density scale and ±0.05 nm regarding the wavelength accuracy. Absorbance spectra were obtained at 298.2 ± 0.1 K in a thermostatic cell holder supplied with a heat transfer module Peltier PTC-2 (PG Instruments, UK). To obtain the absorbance spectra of the BODIPYs, an exact amount of the dye after synthesis, weighed on scales, was dissolved in a 15 ml test tube or in 2 ml of Eppendorf test tube in an exact amount of appropriate solvent to obtain a solution with a known concentration. Then, the test tube was shaken and vortexed until complete dye dissolution was achieved. Next, the aliquot of the solution was transferred to a 1 × 1 cm cuvette, the cuvette was placed into a spectrophotometer, and then the spectra were measured. Extinction coefficients of the dyes in different solvents were obtained similarly by varying the dye's concentration and measuring the absorbance.

*^1^H-NMR.* Proton nuclear magnetic resonance spectra of the dyes were recorded in CDCl_3_ or CCl_4_ (Sigma-Aldrich, USA), depending on the dye type (see Section 1) on an Avance-500 (Bruker, Germany) spectrometer operating at 500 MHz using tetramethylsilane (TMS, analytical standard, for NMR spectroscopy, ACS reagent, Sigma-Aldrich, USA) as an internal reference in the upper Volga region center of the physicochemical research of the G.A. Krestov Institute of Solution Chemistry of the Russian Academy of Sciences. To perform the analysis, the dye was dissolved in CDCl_3_ or CCl_4_ (saturated solutions) and ca. 0.5 ml of a sample was inserted into a 5 mm (outer diameter) 7-inch-long NMR capillary (Wilmad, USA) and closed with a cap. Then, the capillary was placed in a sample changer. Finally, the analysis was carried out via a standard operation manual supplied by the manufacturer.

*Elemental analysis (C, H, N).* Elemental analysis of the synthesized dyes was performed on a FLASH EA1112 (TermoQuest, Italy) elemental analyzer. The analysis starts from the sample preparation; a freshly synthesized BODIPY compound was placed in a desiccator under vacuum for two days to remove the water/solvent residues. Immediately prior to analysis, the sample was removed from the desiccator and was weighed (1–3 mg) in a tin microcontainer on microbalances supplied by TermoQuest with the FLASH EA1112 machine. Then, the container was closed with tweezers, placed into the sampler of the machine, and then analysis proceeded in accordance with the standard operation manual supplied by the manufacturer.

#### 2.3.2 MALDI-TOF Analysis

Matrix-assisted laser desorption/ionization (time-of-flight mass) mass spectra of the synthesized dyes was performed on an AXIMA Confidence MALDI-TOF mass spectrometer (Shimadzu, Japan) equipped with a nitrogen laser (337 nm) in positive ion reflectron mode, using α-cyano-4-hydroxycinnamic acid (CHCA, matrix substance for MALDI-TOF MS, ≥99.0% (HPLC), Sigma-Aldrich, USA). To perform the analysis, two solutions were prepared. Solution 1 (dye solution, 1ml) was prepared in a 2 ml Eppendorf test tube by dissolving BODIPY in 70 : 30 acetonitrile (Sigma-Aldrich, USA): 0.1% trifluoroacetic acid (Sigma-Aldrich, USA) in Milli-Q to reach a concentration of 10 µM. Solution 2 (matrix solution, 1 ml) was prepared in a 2 ml Eppendorf test tube by dissolving 10 mg of CHCA in 1 ml of 70:30 acetonitrile:0.1% trifluoroacetic acid in water. Both solutions were prepared directly prior to analysis, vortexed for complete dissolution, and stored in the dark. Sample application to MALDI-TOF targets was carried out by means of the dried droplet method. In a separate small Eppendorf test tube, 10 µl of solution 1 (the dye solution) was mixed with 10 µl of stock matrix solution 2 and the resulting solution was vortexed. Afterwards, 0.5–2 µl of the resulting solution was applied to the MALDI-TOF target well, and allowed to dry in a sample dryer. Once the solvent completely evaporated, the sample was ready for analysis, which proceeded in accordance with a standard operation manual supplied by the manufacturer.

#### 2.3.3 Dynamic Light Scattering (DLS)

DLS spectra were recorded on a Zetasizer NANO ZSP (Malvern Instruments, UK) instrument at a scattering angle of 173°. Samples for DLS measurements (1 ml) were prepared using the procedures of micellar cluster obtainment and diluted twice with Milli-Q water. All the analyses were carried out in plastic cuvettes for dynamic light scattering (ZEN0118), which have a sample volume of 50 µl. To perform DLS analysis of a pure aqueous micellar solution of TX-114, a TX-114 concentration of ca. 3 mM was introduced into the cuvette and the cuvette was placed into the setup. To perform DLS analysis of clusters comprising mPB, 50 µl of freshly prepared solutions of mPB encapsulated in MCC, mPB-supported MCA, and mPB-based MCC was prepared. Then, in all cases, analysis proceeded in accordance with the standard operation manual supplied by the manufacturer.

#### 2.3.4 pH Measurements

The pH was controlled by preparing a small amount of either a surfactant solution (in a 15 ml test tube) or micellar clusters in an Eppendorf (∼ 0.3–1 ml) test tube; it was measured with a one-electrode pH meter of Eutech Instruments PH700 (Thermo Fischer Scientific, USA). The pH of a TX-114 aqueous solution of 4–11 mM is 5.8–6.1, respectively, at 23 °C, the same that was associated with micellar clusters based on the BPhen metal complex of BODIPY. Prior to analysis, an electrode was washed thoroughly several times with Milli-Q and wiped with lint-free paper. Then the solution for analysis was transferred to a clean small glass beaker and an electrode was put inside the solution. The readings were performed until an equilibrated state of the pH meter was reached. After the measurements, the electrode was cleaned with Milli-Q and returned to the saturated solution of KCl.

#### 2.3.5 Molecular Docking

An automated molecular-docking procedure, using the Autodock 4.2 and (https://autodock.scripps.edu/) MGLTools (https://ccsb.scripps.edu/mgltools/) programs, was performed in order to identify the potential binding sites of compounds on the protein. The Lamarckian genetic algorithm was used to detect the protein-dye conjugate with minimal free energy [13]. Protein was regarded as a rigid molecule; meso-substituent dihedral variation was allowed for the BODIPY molecule. The BSA structure was taken from Protein Data Bank (No. 4F5S) with a charge distribution characteristic for the neutral aqueous media (pH = 7). The initial ligand structures were calculated using the density functional theory method in approximation with the B3LYP hybrid functional and the 6–31G(d, p) basis set using Gaussian W09 software [14]. To estimate the possible interactions of BODIPY with the albumin molecule, at first, “blind docking”, without assigning a specific binding site, was carried out. “Blind docking” calculations were performed for a 97 Å × 97 Å × 97 Å cell with the BSA molecule in the center and a 0.770 Å step. Affinity (grid) Trp213 centered maps of 25.6 Å × 25.6 Å × 25.6 Å grid points and 0.203 Å spacing were used. Each docking experiment was derived from 10 individual runs that were set to terminate after a maximum of 25 million energy evaluations. Complexes of BODIPY and BSA were sorted into groups according to RMS (the root mean square of the coordinates’ difference between this conformation and a cluster reference). For each complex, conformation with minimal energy was assumed to be the most stable. Visualizations of the interactions were performed with the UCSF Chimera package [15].

#### 2.3.6 Fluorescence Spectroscopy

The fluorescence spectra were recorded using a Cary Eclipse spectrofluorometer (Varian-Agilent, USA-Australia; with the detector voltage set to 600 V); Horiba Jobin Yvon Fluorolog 3 spectrofluorometer, USA, 950 V detector voltage); and a SOLAR SM2203 (Belarus) spectrofluorometer. Measurements were carried out in quartz cuvettes having a light-absorbing layer thickness of 1 × 1 cm (for dye solutions) or of 0.1 × 1 mm (for MCCs, a front-face detector was used); emission and excitation slits were varied. All experiments were performed in a thermostatic cell holder at 298.2 ± 0.1 K. The temperature was maintained with the Peltier PTC-2 (PG instruments, UK) heat transfer module. The fluorescence spectra were recorded at a wavelength range of 285–800 nm by varying the excitation wavelengths. To obtain the fluorescence spectra of the BODIPYs, an exact specific amount of the dye after synthesis, weighed on scales, was dissolved in a 15 mL test tube or in 2 ml of Eppendorf test tube in an exact amount of appropriate solvent to obtain a solution with a known concentration. Then, the test tube was shaken and vortexed until full dye dissolution was achieved. Next, the aliquot of the solution was transferred to a 1 × 1 cm cuvette, the cuvette was placed into a spectrofluorometer, and then the fluorescence spectra were measured. The concentration of the BODIPYs was varied to study their aggregation behavior. Fluorescence titration of BODIPY dyes (refer to Figs. 10 and 11) by TX-114 was performed as follows. A 1 × 1 cm cuvette containing a specific BODIPY dye was installed in a cuvette holder in a spectrofluorometer equipped with a magnetic stirrer. Then, an exact volume of 11 mM TX-114 (aqueous solution) was added to the BODIPY solution. The mixture was stirred under ca. 500 RPM for ca. 1 min and then the solution was left for relaxation for ca. 1 min, and finally the spectrum was measured. The procedure was repeated 9 times (refer to Table 2). To perform fluorescence analysis of the dye in pure TX-114 solution, in a supporting state for MCAs and in MCCs (refer to Fig. 12), the procedure of micellar cluster preparation was scaled up to 200 µL to fit a 1 × 10 mm cuvette. In each case, the solution was prepared as described in *“The procedure of micellar cluster preparation” and “Procedures for dye encapsulation in MCC, dye support of MCA, and the dye-based MCC preparation” sections.*

#### 2.3.7 Optical and Fluorescence Microscopy

Fluorescence microscopes were used to obtain microscopy images of the clusters using a Micromed LUM-3, equipped with a ToupCam 5.0 MP CCD digital camera, and an Olympus BX-61, equipped with a QImaging MicroPublisher 3.3 digital camera. To obtain fluorescent images at the UV region, side UV illumination was applied using a UV lamp under a 254/365 nm exposure wavelength (LF-206.LS, UVITec, UK). To perform the optical and fluorescence microscopy measurements, a drop of micellar clusters of any kind was placed onto a piece of glass as described previously in the section on cluster preparation.

#### 2.3.8 Micelle Model Simulation in CHARMM

The process to build a micelle model solvated in water and consisting of TX-114 surfactant molecules was carried out in several steps at the CHARMM-GUI website (http://www.charmm-gui.org). In the first three steps, a micelle-only system was selected from a membrane builder-micelle builder submenu. For the simulation, a rectangular box was used with a water layer thickness (with respect to the surfactant molecule) of 2 nm and no limits for the micelle radius (the program guesses); the output file after this step was uploaded to the Mendeley repository as “Output file after Step 3”. In step four, the option of a lipid ring penetration check was enabled and basic ions (Na^+^ and Cl^–^ in a concentration of 0.1 M) were added using the Monte-Carlo way of placement; the output file after this step was uploaded to the Mendeley repository as “Output file after Step 4”. In step four, energy minimizations were carried out and a water box was added; the output file after this step was uploaded to the Mendeley repository as “Output file after Steps 4-1”. In this step as well, all the components were assembled together; the output file after this step was uploaded to the Mendeley repository as “Output file after Step 5”. In Step 6, the CHARMM36m force field was selected as well as hydrogen mass repartitioning and a WYF parameter for cation-pi interactions and a temperature of the system was set to 298.15 K; the output file after this step was uploaded to the Mendeley repository as “Output file after Step 6”. After the output, a *.PDB file was generated and the structure of the micelle was visualized using the ChimeraX 1.4 package (https://www.cgl.ucsf.edu/chimerax/). Several modes of visualization were applied, with/without water molecules and counter-ions. All of the datasets generated by CHARMM-GUI for the input/output files after every available step were uploaded to a Mendeley repository.

## Ethics Statements

This article does not involve any studies with human participants, animals, or data collected from social media platforms.

## CRediT authorship contribution statement

**Aleksei V. Solomonov:** Conceptualization, Methodology, Validation, Formal analysis, Investigation, Resources, Writing – original draft, Writing – review & editing, Visualization, Project administration. **Yuriy S. Marfin:** Investigation, Resources, Writing – original draft, Funding acquisition. **Alexander B. Tesler:** Writing – original draft. **Dmitry A. Merkushev:** Investigation. **Elizaveta A. Bogatyreva:** Investigation. **Elena V. Antina:** Investigation, Resources, Writing – original draft. **Evgeniy V. Rumyantsev:** Resources, Funding acquisition. **Ulyana Shimanovich:** Writing – review & editing.

## Declaration of Competing Interest

The authors declare that they have no known competing financial interests or personal relationships that could have appeared to influence the work reported in this paper.

## Data Availability

Dataset for the Synthesis of Boron-Dipyrrin Dyes, their Fluorescent Properties, their Interaction with Proteins, Triton-X-based surfactants, and Micellar Clusterization Approaches to Validation based (Original data) (Mendeley Data).
